# A Multimethod Evaluation to Assess Feasibility, Acceptability, and Preliminary Efficacy of HPVVaxFacts, a Tailored Mobile Web App, for Parents With Unvaccinated Children: Pilot 2-Arm Randomized Controlled Trial

**DOI:** 10.2196/78910

**Published:** 2026-09-09

**Authors:** Jennifer Cunningham-Erves, Pamela C Hull, Amanda F Dempsey, Tatsuki Koyama, Lili Sun, Mariya Chalise, Elizabeth C Stewart, Jessica L Jones, Douglas Landsittel, Meredith Smalls, Freneka F Minter, Lindsay S Mayberry, Lora Harnack, Michael J Hook, Janet Cates, Tammy Van Maanen, Taylor Burnett, Consuelo H Wilkins

**Affiliations:** 1 Department of Health Policy, Vanderbilt University Medical Center, 2525 West End Avenue, Suite 700, Nashville, TN, United States, 615-541-9375; 2 Behavioral Science Department, University of Kentucky, Lexington, KY, United States; 3 MERCK, Rahway, NJ, United States; 4 Department of Biostatistics, Vanderbilt University Medical Center, Nashville, TN, United States; 5 Graduate School of Public Health Practice, Meharry Medical College, Nashville, TN, United States; 6 Meharry-Vanderbilt Alliance, Nashville, TN, United States; 7 Department of Biostatistics, The State University of New York, University at Buffalo, , Buffalo, NY, United States; 8 Department of Medicine, Vanderbilt University Medical Center, Nashville, TN, United States; 9 Cumberland Pediatric Foundation, Nashville, TN, United States; 10 Spring Creek Pediatrics, Chattanooga, TN, United States; 11 Department of Family Medicine, Meharry Medical College, , Nashville, TN, United States

**Keywords:** human papillomavirus, vaccine uptake, digital intervention, mHealth, children and adolescents, parents, HPV, mobile health

## Abstract

**Background:**

Mobile health (mHealth) interventions may improve provider-parent communication on human papillomavirus (HPV) vaccination to reduce concerns, and increase intention and uptake. HPVVaxFacts (233 Analytics) is a novel, mobile web app delivering tailored education based on the Health Belief Model and Theory of Reasoned Action, addressing parental concerns preclinic visit.

**Objective:**

This study aimed to assess the feasibility, acceptability, and preliminary efficacy of HPVVaxFacts among parents of adolescents aged 9‐17 years.

**Methods:**

We conducted a pilot, randomized controlled trial in 2 urban Tennessee clinics from June to September 2023 comparing 2 groups: tailored education via HPVVaxFacts mobile web app (intervention, n=27), and nutrition education (attention control, n=30). Eligible parents had or were caregivers to a child aged 9 to 17 years unvaccinated against HPV, had a mobile phone, had an upcoming clinic visit, and spoke English. The recruitment strategy was patient intake software—Phreesia (Phreesia, Inc) and eClinicalWorks (eClinicalWorks). Although unblinded, parents could deduce their study arm assignment. Providers were blinded. Feasibility, acceptability, and preliminary efficacy (HPV vaccine knowledge, concerns, intentions, and vaccination rates) were assessed using multimethod evaluation. Parents were assessed at baseline and immediately post intervention via surveys. Vaccination rates were assessed at 12 months post intervention via electronic health records. Nineteen parent interviews were conducted up to 9 months post intervention. A clinic staff consultation (n=6) was 1 month post intervention.

**Results:**

Of 57 enrolled parents, most were female (52/57, 91%), non-Hispanic White (44/57, 77%), had ≤US $80,000 household income (32/57, 56%), and had some college or less (27/57, 47%). In total, 81% (29/36) of parents viewed HPVVaxFacts. Post intervention, HPV vaccine initiation was higher in the intervention group compared to the attention control group (48% vs 17%; difference 0.24; 95% CI 0.03-0.46; *P*=.01). Parents in the HPVVaxFacts arm demonstrated a greater reduction in knowledge (ie, knowledge increase; mean change: −0.6 vs 0.1) and concern scores (mean change: −3.4 vs −1.4) than those in the nutrition education arm. However, between-arm differences were not statistically significant (*P*=.13 and *P*=.14, respectively). The majority found the study protocol and HPVVaxFacts acceptable. Benefits of HPVVaxFacts include confirming their decision to vaccinate, supporting parent-child discussion on the vaccine, and answering questions preclinic visit or offering questions for the provider. Study protocol delivery and mobile web app instructions were suggested areas for improvement. Barriers for HPVVaxFacts use include content in English only and digital format.

**Conclusions:**

Our study suggests HPVVaxFacts was feasible and acceptable among parents to provide previsit, tailored information on HPV vaccination. Outcomes offer a positive trajectory but need more exploration. Next steps include a well-powered efficacy trial to determine the impact of HPVVaxFacts on initiation vaccine rates and parental hesitancy factors, as well as to explore an interaction, effect modification, and mediation among different variables.

## Introduction

Parental vaccine hesitancy remains a critical barrier to suboptimal rates of human papillomavirus (HPV) vaccination. Studies show up to 63% of US parents are hesitant [1,2] (ie, reluctant or refuse to vaccinate) despite its availability and proven effectiveness [3]. This contributes to stalled HPV vaccination rates among adolescents aged 13‐17 years, 63% in the United States and 64% in Tennessee in 2025 [4]. Unvaccinated adolescents are at risk for HPV infection, which is implicated in almost all cervical cancers and 63%‐90% of penile, vulvar, vaginal, anal, and oropharyngeal cancers [5]. Many of these HPV-related cancers are rising in incidence [6]. Based on several US studies, parental predictors for HPV vaccine hesitancy are complex and multifactorial [7-9]. Common reasons include concern about safety or side effects, lack of knowledge on HPV and associated cancers, and lack of or poor-quality provider recommendations and communication [10]. Hesitant parents often report dissatisfaction with patient-provider communication, further undermining their confidence and decision-making to initiate and complete HPV vaccination [11-14]. There is an urgent call to identify evidence-based practices (EBPs) to address vaccine hesitancy globally and nationally [4,13,15].

Few interventions to date have been found impactful in addressing parental HPV vaccine hesitancy. Mobile health (mHealth) interventions show promise in enhancing traditional medical care. With provider-based strategies (eg, a strong recommendation and motivational interviewing), mHealth interventions could positively impact parental concerns by countering misinformation or addressing lack of information about HPV vaccination [16-22]. Some data exist on mHealth in promoting HPV vaccination, but important limitations remain. A systematic review from January 2017 to July 2022 identified 24 digital health interventions for HPV vaccination, with only 4 using theory-based mHealth interventions [23-26]. Limitations of these interventions are (1) a focus on general HPV vaccine promotion and not unique concerns of vaccine-hesitant parents, (2) targeted only at girls or younger adolescents, and (3) limited testing across diverse sociodemographics and geography. Two of these studies tailored information using reminders or by individual vaccine status [23,26-29], but these approaches were limited in impact. There remains an unmet need to develop an efficacious mHealth program that provides parents succinct, resonating messages individually tailored to their concerns on the HPV vaccine for use by providers to supplement parent-provider in-clinic discussion [30,31].

Past studies suggested that many parents wanted HPV vaccine information before a clinic visit with their child’s provider. Information could be delivered prior to the clinic visit using mHealth [16-22]. The purpose of receiving this information was to get the answers to the questions they may have about HPV and the vaccine prior to the visit. Furthermore, parents used this information to engage in parent-provider communication about the vaccination and support shared decision-making (J Cunningham-Erves, unpublished data, June 2022).

This study’s objective was to conduct a pilot test of a mobile web app intervention, HPVVaxFacts (233 Analytics), designed to provide tailored, previsit education to improve HPV vaccination rates. We hypothesized that : (1) the HPVVaxFacts intervention arm would achieve acceptability and feasibility rates ≥70%; and (2) parents in the intervention arm would show greater reductions in vaccine concerns and greater increases in knowledge, intentions, and vaccination rates compared to the attention control arm. To prepare for a future full-scale randomized controlled trial (RCT) to determine efficacy, our aims were as follows:

- Aim 1: To assess feasibility of the HPVVaxFacts intervention and overall study protocol.
- Aim 2: To assess acceptability of the HPVVaxFacts intervention within 2 urban pediatric clinics.
- Aim 3: To explore preliminary efficacy of the HPVVaxFacts intervention on the parent’s decision to get their adolescents the HPV vaccination.

## Methods

### Setting and Study Design

A multimethod approach was used to evaluate the feasibility, acceptability, and preliminary efficacy of a mobile web app, HPVVaxFacts, among parents of unvaccinated children aged 9 to 17 years [32]. A pilot, 2-arm, multisite RCT was conducted at 2 urban pediatric clinics in Tennessee to determine feasibility and acceptability. Clinic Site 1 is an urban pediatric clinic serving more than 8000 children with a broader demographic of families with varied socioeconomic backgrounds. There were 421 unvaccinated children at Site 1 from June to September 2023. In contrast, Clinic Site 2 is an urban pediatric clinic serving more than 5000 children primarily uninsured, underinsured, and marginalized children. A total of 23 unvaccinated children were at Site 2 for 1 week in August 2023. Clinic providers and staff stated they had higher vaccination rates, so they chose a 1-week back-to-school event where vaccinations were offered. This time was perceived to have the highest volume of unvaccinated patients. While operational workflows (ie, administrative protocols, patient volume, and technology adoption) and catchment areas differ, both had patient intake management platforms for recruitment and followed the standardized protocols to be discussed in detail below.

Parents in both arms who agreed to follow-up were interviewed to explore acceptability and feasibility of the protocol using a qualitative, descriptive study design. Intervention acceptability and parent-child discussion were examined among parents in the intervention arm only. The reporting of the pilot RCT follows the CONSORT (Consolidated Standards of Reporting Trials) 2010 guidelines to report a pilot or feasibility trial (Multimedia Appendix 1) and the CONSORT-EHEALTH (Consolidated Standards of Reporting Trials of Electronic and Mobile Health Applications and Online Telehealth) checklist to evaluate web-based apps (CONSORT-HEALTH checklist in Checklist 1) [33].

### Ethical Considerations

The Institutional Review Board (IRB) of Meharry Medical College (Protocol Number: 18-12-890) approved this study as expedited as it was considered no more than minimal risk. The study was registered at ClinicalTrials.gov (NCT04380623) and performed in line with the principles of the Declaration of Helsinki. Electronic consent was obtained from participants using REDCap (Vanderbilt University) [34,35] for the pilot RCT, and verbal consent for the semistructured interviews. To protect participant information, survey and interview data were stored via REDCap and on a password-protected server. Data were deidentified prior to provision to statisticians and research team members for data analysis. Dissemination of results was aggregate and anonymous. Participants were compensated with a US $30 gift card for pilot study participation, and a US $20 gift card for completing a follow-up interview.

### Intervention

**Figure 1.**
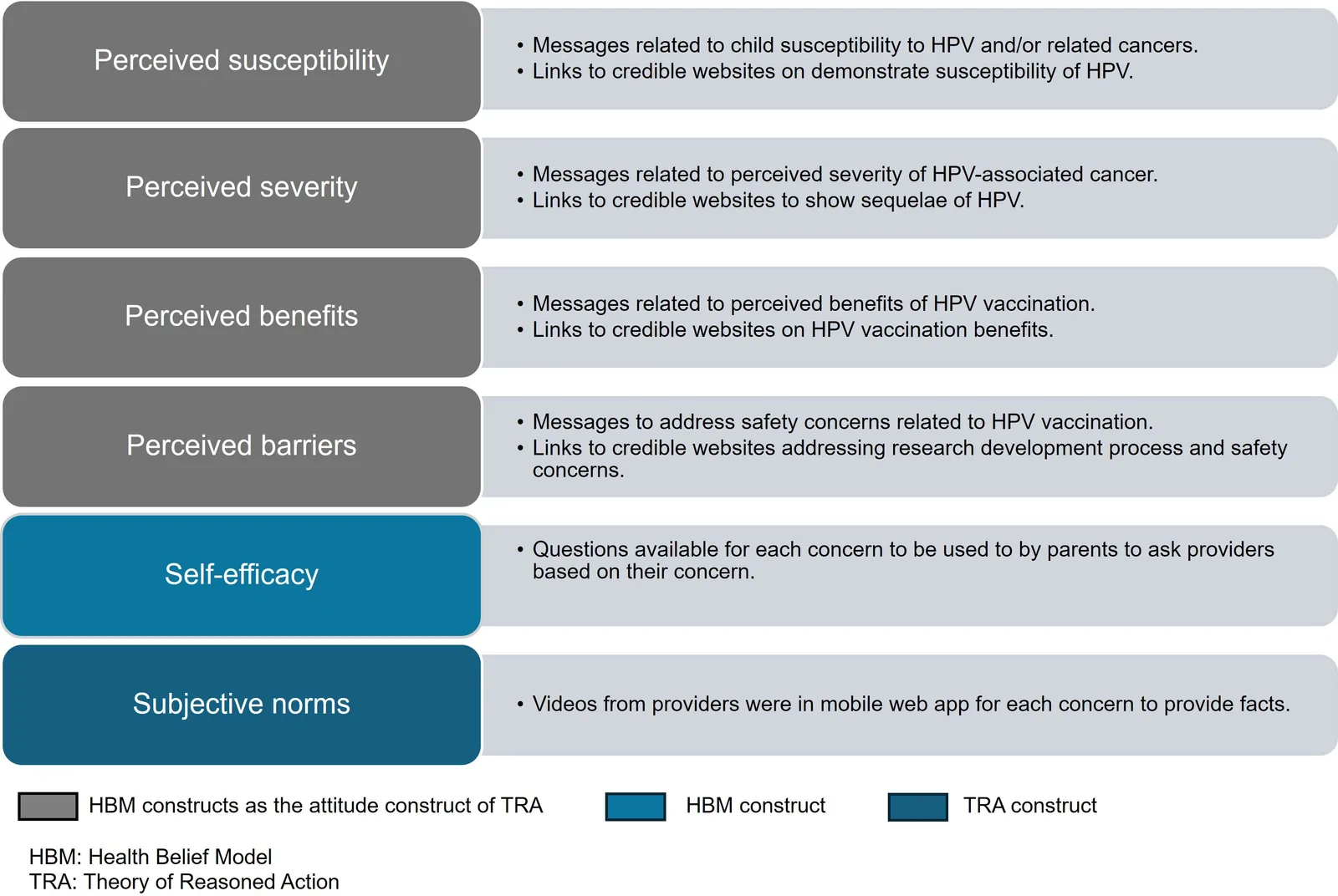
Examples of theory application in HPVVaxFacts development. HPV: human papillomavirus.

HPVVaxFacts provides individually tailored education on HPV and the vaccine to parents [36]. Education and video content for each concern was developed using the Health Belief Model and Theory of Reasoned Action (Figure 1). To tailor the education in the app, each parent answered a 10-item baseline survey adapted from the Vaccination Confidence Scale [37] to identify parental concerns. The survey responses are mapped to an algorithm that selects the top 3 parent concerns. Parents can change the selection if they disagree. Parents then review the content (ie, educational information, provider’s video, questions for the provider, and links to other credible sources verifying content) associated with each concern. An adolescent corner is available for parents to share with and/or have an optional discussion with their child about each concern. Finally, parents could submit concerns or additional needs via the provision of feedback. Refer to Cunningham-Erves et al [36] for full description of HPVVaxFacts development.

### Attention Control

Parents in the attention control arm received a link to a website providing nutrition education for parents of children and/or teens [38], a program unrelated to the study outcome. This approach served two purposes: (1) ensuring all parents receive a benefit regardless of study arm assignment, and (2) balancing time and effort between groups [39]. Because parents’ perceptions of HPV vaccination significantly influence vaccination outcomes [40], an attention control group allowed us to account for potential placebo effects and isolate the specific effects of HPVVaxFacts. Furthermore, the study arm activities were comparable to the intervention activities. Parents completed a 5-item brief quiz with immediate feedback on correct or incorrect responses and explanations.

### Study Participants

This study was conducted with parents who were caregivers for children in 2 urban pediatric clinics in Tennessee who were representative of the continuum of vaccine hesitancy. Eligible parents had a child aged 9 to 17 years who was unvaccinated against HPV, had a mobile phone, had an upcoming clinic visit, and spoke English. When multiple children per eligible caregiver existed, random selection determined which child would participate. Random numbers were first generated in Stata version 17 (StataCorp LLC). Children were ordered from youngest to oldest (or alphabetized by first names if twins or triplets). The child with the lowest corresponding random number was selected for the parent to be sent a study invitation.

Eligibility criteria were chosen based on participating providers and staff input. They stated it was not feasible to enroll only hesitant parents. Instead, they perceived it was equally beneficial to recruit parents representing the continuum of hesitancy. Exclusion criteria were parents who did not consent, or their child had initiated or completed the HPV vaccination series.

### Multimethod Evaluation

#### Quantitative Phase

##### Training and Preparation

The clinic sites had different recruitment approaches. Clinic Site 1 recruitment was conducted by 2 trained clinic staff. Clinic Site 2 recruitment was done by 2 members of the research team. Recruitment and enrollment procedures were standardized through 2-hour training. Training included a review of the study protocol and documents, integration with clinic workflow, and potential recruitment and retention challenges. Post training, a study protocol run-through was conducted at each clinic site to familiarize the study team with the protocol and identify necessary adjustments prior to implementation.

##### Site-Specific Recruitment Methods

At Clinic Site 1, parents were first introduced to the study via a post to the clinic’s Facebook (Meta Platforms, Inc) page to increase study awareness in their clinic. At Clinic Site 2, flyers were posted in the clinic. Eligibility was determined via electronic health record (EHR) data by 2 trained clinic staff at Clinic Site 1 and 2 trained research staff at Clinic Site 2. The research team was trained by clinic staff to use EHR and eClinicalWorks (version 12.0.2; eClinicalWorks).

##### Enrollment Communication

Eligible parents received an IRB-approved text message explaining the study and inviting study participation, with emphasis on the HPV vaccination. If parents text “yes,” a screener was sent text to parents to confirm eligibility. Trained clinic staff at Clinic Site 1 contacted parents via Phreesia, a HIPAA (Health Insurance Portability and Accountability Act)-compliant patient intake management platform that automates the check-in process as well as supports patient-provider communication [41]. At Clinic Site 2, parents were contacted via eClinicalWorks by study team members. Parents received up to 3 text messages on their mobile phones requesting participation.

##### Sample Size

GPower 3.1 program (version 3.1.9.7; Heinrich-Heine-Universität Düsseldorf) was used to conduct power analysis to determine the sample size. The necessary sample size for the pilot trial was calculated using a 1-sided 80% CI and the expected effect size of the future RCT [42], based on the expected proportion in the attention control arm (0.20‐0.30) and the clinically meaningful difference to be observed in the intervention arm in the future RCT (at least 0.10 higher) [42,43]. A total pilot sample size of 46‐60 accounts for expected attrition and was deemed sufficient. Our study goal was to enroll 70 parents, expecting 10%‐15% attrition (eg, no-show for clinic visit), yielding a final sample of 60‐63 parents (28‐30 per arm).

##### Randomization and Blinding

All data were collected via the parents’ mobile phones. A member of the research study (coauthors: JC-E, ECS, and MS), all trained and with social science backgrounds, enrolled parents in the study. The biostatistician (coauthor DL) generated 2 randomization lists prior to study implementation. Using blocked randomization, randomization was conducted with blocks of 10 (using n=10 from each group). Group labels (denoted as 1 or 2) were generated in Stata version 17. Although parents could deduce their study arm assignment, providers were blinded. We had 2 protocol deviations in which 1 participant who decided not to participate later decided to participate, which led to 2 parents being assigned to the HPV program at the same time. We decided to retain both participants for the analysis. The second deviation was the continued enrollment of 7 participants more than the planned sample size due to consent initiation. However, these individuals were not a part of the random allocation process and were excluded [44].

##### Procedures

After confirming participant eligibility, parents were sent a REDCap link to their mobile phones to provide electronic consent up to 48 hours prior to the clinic visit. They were assigned to either the intervention or attention control arm post consent based on the computer-generated randomization numbers listed by a trained research team member with a social science background (lead author: JC-E). Parents were asked to view HPVVaxFacts or nutrition programs at least once prior to their upcoming clinic visit. At baseline, parents took the presurvey assessing their sociodemographics and knowledge and concerns related to HPV and the vaccine, and intentions to initiate vaccination. If randomized to the intervention arm, parents were sent a personalized link to the presurvey to assess knowledge and intention and then went to the mobile web app HPVVaxFacts to assess, receive, and confirm or change their top 3 concerns. After viewing the assigned mobile web app, parents took a postsurvey before the clinic visit with similar presurvey questions except for the sociodemographics. Parents in the intervention arm were also asked questions related to program acceptability. If any participant did not complete a survey or view the mobile web app, up to 3 reminders were sent for completion. Post study, the trained clinic staff provided a dataset on the child’s HPV vaccine status at 0 and 12 months.

##### Outcomes

###### Overview

The clinic provided the vaccination status of each child at 12 months post intervention using the EHR (Primary Outcome). The pre-post survey asked parents about their knowledge, concerns, and intentions for their child to initiate the HPV vaccination process. Intervention acceptability and intervention use for parent-child discussion were assessed on the postsurvey of parents in the intervention, “HPVVaxFacts” study arm only. Intervention feasibility was assessed via several feasibility indicators (Secondary Outcomes). Sociodemographic data were collected on the presurvey only (Covariates).

###### Primary Outcome: HPV Vaccination Status (1-Item)

HPV vaccination status post study was retrieved from the clinics’ EHR system. The child’s vaccination status post study was labeled as “yes*”* and *“*no.*”* Vaccination status was assessed at 12 months post intervention.

###### Secondary Outcomes

####### Knowledge of HPV and the Vaccine (10-Item Scale)

A validated knowledge scale [45] assessed parental knowledge of HPV and HPV vaccination. Response options were true, false, or unsure, in which some answers were true and others false. For analysis, false responses were reverse-coded, false and unsure answer responses were combined, and mean scores were calculated for pre- and postassessments. The lower the score, the greater the degree of knowledge a parent had. When measuring internal consistency reliability, the Cronbach α for knowledge data on presurvey was 0.80 and 0.81 on the postsurvey.

####### Concerns (9-Item Scale)

An adapted attitudes scale, the Vaccine Confidence Scale [37], assessed parental concerns on HPV vaccination based on a 4-point Likert scale to determine agreement. The parents receiving nutrition education completed this measure in REDCap, while parents receiving HPVVaxFacts completed these items in the mobile web app. The HPVVaxFacts mobile web app prompted parents to take a survey, receive their top 3 concerns based on a preexisting algorithm, and confirm and/or change their selection. The final selected top concerns were tracked within the app. For inferential analysis, mean pre- and postscores were calculated for both groups with the greater score indicating more parental vaccine concerns. Internal consistency was strong: Cronbach α 0.90 (presurvey) and 0.91 (postsurvey).

####### HPV Vaccination Intention (1-Item)

One question asked, “How likely is it that you will get your child the HPV vaccine?” with the response options “Definitely not likely,” “Not likely,” “Somewhat likely,” and “Very likely” [46].

####### HPV Vaccine Recommendations

Parents were asked, “Has your child’s doctor offered the HPV vaccine?” with answer options yes or no.

####### HPVVaxFacts Feasibility

We followed guidelines of Teresi et al [47] to assess various aspects of feasibility in our pilot study. We describe each below.

### Data Collection Protocols and Data Availability

We documented whether participants complied with assessment and data collection protocols. This included (1) completion rates of the pre-post survey, (2) mobile web app tracking of parent concerns and selected top 3 concerns by date and time, and (3) the ability to obtain the necessary data from clinic administrative records, particularly age and HPV vaccination status. Feasibility was measured as 70% of parents viewing the mobile web app.

### Conceptual and Psychometric Adequacy of Measures

We assessed the internal reliability of the concerns, knowledge, and intervention acceptability scales. Participants were allowed to provide feedback on the HPVVaxFacts mobile web app to determine whether important concerns were missing from the app, which suggests missed questions on the concern scale.

### Intervention Implementation

We assessed the number of participants recruited and retained for the study.

#### HPVVaxFacts Acceptability (4-Items)

Parents were asked their acceptability of HPVVaxFacts using a validated, 4-item scale. Response options were on a 5-point Likert scale based on agreement [48]. An example item is “The HPV Education Program meets my approval.” Acceptability was measured as 70% or more of parents stating they agree or strongly agree with each statement. Internal consistency yielded the Cronbach α value of 0.91 on presurvey data in this study.

#### Parent-Child Discussion (1-Item)

Developed by the research team, parents in the intervention arm were asked whether HPVVaxFacts was helpful in discussing the HPV vaccine with their child post study. The answer choices were on a 5-point Likert scale based on agreement.

### Covariates: Sociodemographics (12-Items, Presurvey Only)

These variables were in 2 categories—parent and child sociodemographics.

### Parent Sociodemographics

Age was a continuous variable. Sex was categorized as male, female, and prefer not to disclose. Parent sexual orientation included categories of asexual, bisexual, straight, fluid, and gay. Categories were combined to straight (heterosexual), prefer not to disclose, and other. Parents’ race or ethnic categories were non-Hispanic White, African American, Latino or Spanish origin, Native Hawaiian or Other Pacific Islander, and Prefer Not to Answer. Education categories were General Educational Development (GED) or high school diploma, associate’s degree, some college, bachelor’s degree, master’s degree*,* and doctoral or professional degree. Annual household income categories were less than US $20,000, US $20,001 to US $40,000, US $40,001 to US $60,000, US $60,001 to US $80,000, and more than $80,000, or did not answer.

### Child Sociodemographics

Age, a continuous variable, was categorized to 9‐10, 11‐12, and ≥13. Sex was categorized as male, female*,* and prefer not to disclose. The child’s race or ethnic categories were non-Hispanic White, African American, Latino or Spanish origin, American Indian and Alaskan Native, Prefer Not to Answer, and Other. The child’s sexual orientation had several categories including aromantic, asexual, bisexual, fluid, straight, and gay. These categories were combined to straight (heterosexual), questioning or unsure, prefer not to disclose, and other*.*

### Data Analysis

Descriptive statistics characterized the sample: continuous variables are reported as means and SDs, and categorical variables as frequencies and percentages. Between-group comparisons were conducted using appropriate statistical tests: Fisher exact test or chi-square tests for categorical variables and Wilcoxon rank-sum tests or *t* tests for continuous variables. Comparison of pre-post scores of HPV vaccine knowledge, concerns, and intention by study arm was performed using exploratory visual analysis. Provider recommendation was not included as a covariate because (1) recommendations were expected to be similar across study arms and (2) are rarely systematically documented in clinical practice [49], making reliable data collection impractical. Parents took the survey in the HPVVaxFacts mobile web app and were allowed to select their top concerns. The research team categorized the top 3 concerns and summed the concerns by question. The summed scores were sorted from highest to lowest, with the top 3 values being the top concerns.

### Qualitative Phase

#### Recruitment

Interviews were conducted up to 9 months post intervention based on parent availability. On the postsurvey, 15 in the intervention study arm and 18 in the nutrition attention control agreed to be contacted for follow-up interviews. Of these participants, 9 in the intervention study arm and 10 in the nutrition attention control completed an interview. Other parents either could not be reached (n=9) to complete or later decided not to complete the interview (n=5).

#### Procedures

Parents in both intervention and attention control groups who agreed to be contacted for the follow-up interview were called to schedule the 20-minute interview. The purpose of these interviews was to complement quantitative data. Interviews were audio-recorded and transcribed for data analysis.

#### Interview Protocol

The semistructured interview protocol for parents who were assigned to the HPVVaxFacts intervention arm included questions that explored intervention acceptability. Parents in both study arms were queried regarding their study experiences and ways to improve the study protocol and implementation procedures.

#### Data Analysis

Interview transcripts were analyzed using a modified version of Braun and Clarke’s 6-step thematic methodology [50]. A trained qualitative researcher and analyst (coauthors JC-E and TB) conducted the data analysis led by lead author JC-E, both with backgrounds in social science. Using the first 3 interview transcripts, coauthor JC-E created a codebook of a priori codes using line-by-line coding. Both coders used the codebook to independently code each transcript, creating reliability in the system and having each coder serve as a referent. The analysts then met to review coding decisions and reach a consensus on whether to accept, modify, remove, or add codes until a percentage agreement of >90% was reached. Coding saturation was met when no new codes emerged. Patterns and themes were generated by combining related codes and applying constant comparison methods across transcripts. Rigor was established using thick, rich descriptions of interview data. Interview data were managed using Excel (version 26; Microsoft 365).

### Clinic Staff Feedback From Consultation

One month post intervention (October 2023), we held a 1-hour consultation (ie, an engagement strategy) with 2 clinic providers and 4 staff members over lunch to gather informal feedback on study protocol and implementation. Trained clinic staff coordinated the date and time of the consultation. The consultation explored ways to improve the study protocol and implementation procedures, and perceived barriers and facilitators to implementing HPVVaxFacts in routine clinic practice. This session was not audio-taped nor formally analyzed as qualitative research; instead, notes were recorded by coauthors JC-E and ES.

Overall, providers perceived that the program offered clinic benefits. Offering education preclinic visits to their patients was a top benefit. This complemented their recommendations and lessened clinic time for education. Another perceived benefit was the increased vaccination rates in the clinic. Regarding barriers, providers and clinic staff highlighted the need to improve logistics for large-scale implementation. Because the intervention was manual and required efforts from clinic staff and the research team to aid in recruitment and retention, providers questioned the feasibility of this study without full integration into the EHR but still perceived offering preclinic information in general as feasible. However, providers and staff stated they understood this was a pilot study and hoped their suggestions improved future implementation.

## Results

### Feasibility: Recruitment and Retention

**Figure 2.**
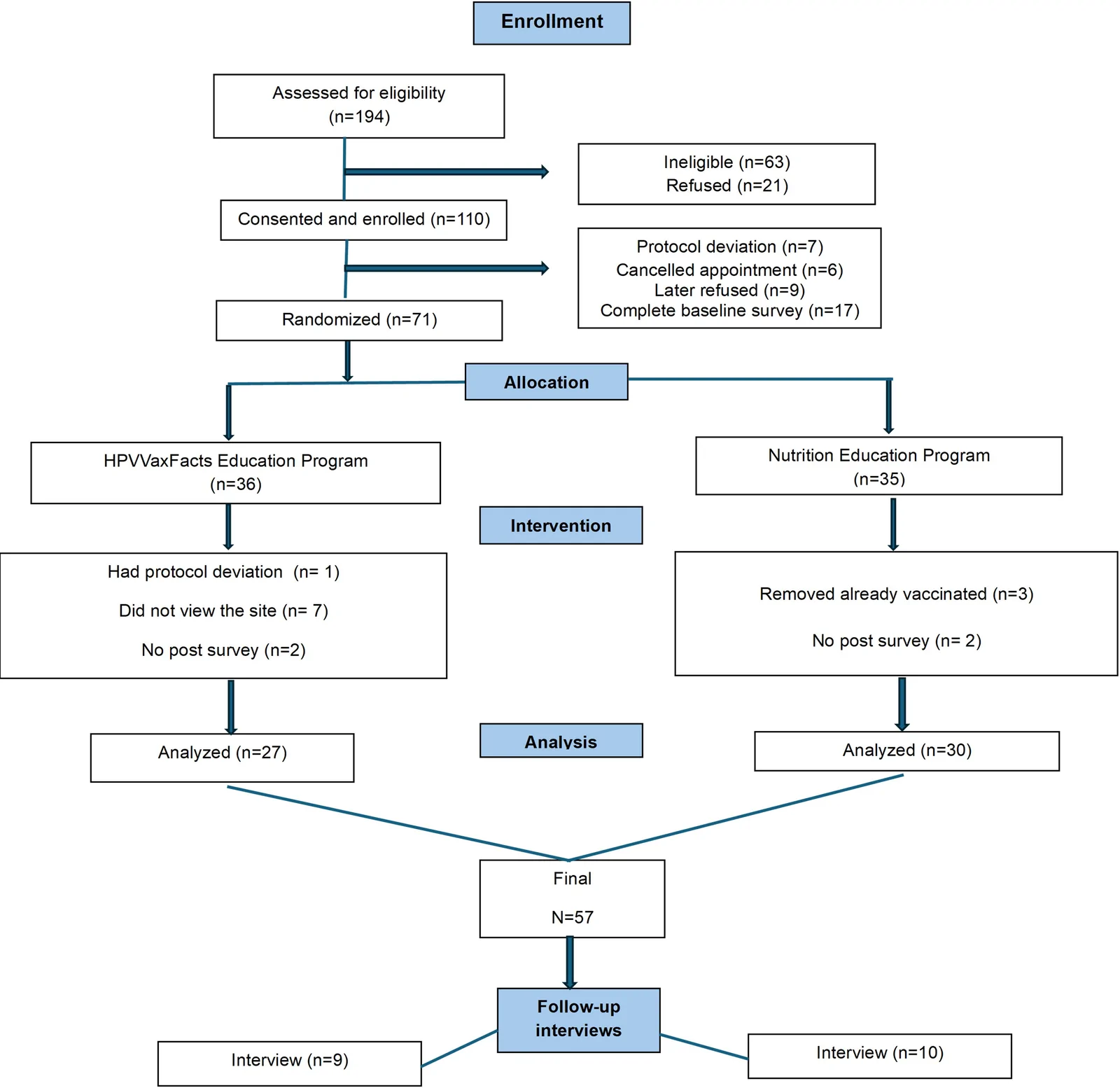
CONSORT (Consolidated Standards of Reporting Trials) flow diagram.

We met our goal of training 2 clinic staff to aid in recruiting participants through Phreesia. For this study, the clinic staff sent potential parents an IRB-approved REDCap link with a screener. If they screened eligible, they received the presurvey, viewed the intervention, and took the postsurvey. Of the 194 assessed for eligibility, 63 were ineligible. A total of 21 (16%) declined to participate and 9 more later refused, increasing the refusal rate to 23%. We recruited 110 participants who consented and enrolled in the study (107 were recruited from Clinic Site 1 and 3 from Clinic Site 2) with an enrollment rate of 84%. Parents were enrolled 15 to 20 minutes up to 24 hours prior to the clinic visit. In total, 71 people were allocated to the study arms, a 35.5% reduction from enrollment to randomization. A total of 36 parents were randomly assigned to the HPVVaxFacts intervention arm, of which 7 did not go to the mobile web app. Of those who viewed the mobile web app (81%), 2 did not complete the postsurvey. These participants were excluded with a retention rate of 75%. A total of 35 people were randomly assigned to the attention control arm. We were unable to determine whether participants viewed the nutrition education website. However, 2 did not complete the postsurvey with a retention rate of 94% (Figure 2).

### Quantitative: Child and Parent Characteristics

The sociodemographic and clinical data by study arm assignment reported by parents demonstrated that most children belonged to the 11‐12 age group with 44% and 50% in the HPVVaxFacts and nutrition study arms, respectively. Children in both groups were predominantly female, non-Hispanic White, and heterosexual. Among parents who participated in the survey, most of them were heterosexual. The mean age for participants in the HPVVaxFacts intervention arm was 41.8 (SD 14.6) years compared to participants in the nutrition attention control arm at 41.6 (SD 7.5) years. In terms of education level, a higher proportion of the parents in the HPVVaxFacts intervention arm had a bachelor’s degree or higher (18/27, 66%) than those in the nutrition attention control arm (11/30, 40%). Additionally, the majority in both arms reported an annual household income of more than US $60,000. In the HPVVaxFacts intervention arm, 59% (16/27) of parents had been offered the HPV vaccine for their child by a provider compared to 70% (21/30) in the nutrition attention control arm. More than half in both arms were iPhone users (Table 1).

**Table 1.** Demographic and clinical information of parent and their child (N=57).

Characteristics	HPVVaxFacts program (n=27)	Nutrition program (n=30)
Child age (years), n (%)		
9‐10	7 (26)	4 (13)
11‐12	12 (44)	15 (50)
≥13	8 (30)	11 (37)
Child sex, n (%)		
Male	8 (30)	12 (40)
Female	19 (70)	18 (60)
Prefer not to answer	0 (0)	0 (0)
Child sex identification, n (%)		
Straight (heterosexual)	24 (89)	25 (83)
Questioning or unsure	0 (0)	1 (3)
Prefer not to disclose	3 (11)	3 (10)
Additional category or identity not listed	0 (0)	1 (3)
Child race, n (%)		
Non-Hispanic White	19 (70)	24 (80)
African American, African, or Afro Caribbean	5 (19)	3 (10)
Latino or Spanish Origin	1 (4)	0 (0)
American Indian, Alaskan Native, Native American	0 (0)	1 (3)
Native Hawaiian or Other Pacific Islander	0 (0)	0 (0)
Prefer not to answer	1 (4)	1 (3)
Other	1 (4)	1 (3)
Parent age (years), mean (SD)	41.8 (14.6)	41.6 (7.5)
Parent sex, n (%)		
Male	2 (7)	1 (3)
Female	23 (85)	29 (97)
Prefer not to answer	2 (7)	0 (0)
Parent sex identification, n (%)		
Straight (heterosexual)	25 (93)	29 (97)
Questioning or unsure	0 (0)	0 (0)
Prefer not to disclose	1 (4)	1 (3)
Additional category or identity not listed	1 (4)	0 (0)
Parent race, n (%)		
Non-Hispanic White	19 (70)	25 (83)
African American, African, or Afro Caribbean	5 (19)	2 (7)
American Indian or Alaskan Native	0 (0)	0 (0)
Latino or Spanish Origin	1 (4)	2 (7)
Native Hawaiian or Other Pacific Islander	1 (4)	0 (0)
Prefer not to answer	1 (4)	1 (3)
Parent education, n (%)		
GED^a^ or High School Diploma	3 (11)	6 (20)
Associate’s degree	5 (19)	8 (27)
Some college	1 (4)	4 (13)
Bachelor’s degree	12 (44)	6 (20)
Master’s degree	3 (11)	5 (17)
Doctoral or Professional degree	3 (11)	1 (3)
Household income (US $), n (%)		
<$20,000	2 (7)	1 (3)
$20,001 to $40,000	4 (15)	3 (10)
$40,001 to $60,000	4 (15)	3 (10)
$60,001 to $80,000	7 (26)	8 (27)
>$80,000	9 (33)	12 (40)
Did not want to answer	1 (4)	3 (10)
Phone type, n (%)		
Android	9 (33)	8 (27)
iPhone	18 (67)	22 (73)
Offered HPV^b^ vaccination for child, n (%)		
Yes	16 (59)	21 (70)
No	11 (41)	9 (30)

a GED: General Educational Development.

b HPV: human papillomavirus.

### HPV Vaccine Initiation Status Poststudy by Study Arm

HPV vaccine status was obtained for 57 children aged 9‐17 years who were nonvaccinated for HPV prior to their parent participating in the clinical trial. HPV vaccine initiation (ie, those who received the first dose) rates of children whose parents participated in the HPVVaxFacts intervention arm were significantly higher than those of children whose parents were assigned to the nutrition attention control arm (48% vs 17%; Table 2).

**Table 2.** HPV^a^ vaccine rates 12 months poststudy by study arm (N=57).

	HPVVaxFacts Intervention (n=27)	Nutrition Attention Control (n=30)	Total (N=57)	*P* value
Received >1 dose of HPV vaccine, n (%)				.01
Yes	13 (48)	5 (17)	18 (32)	
No	14 (52)	25 (83)	39 (68)	

a HPV: human papillomavirus.

### Factors Associated With HPV Vaccination by Study Arm

We determined the impact of the HPVVaxFacts intervention and nutrition attention control on knowledge, concerns, and intention related to HPV vaccination. Analysis revealed that parents in the HPVVaxFacts intervention arm showed higher improvement in knowledge scores but remained largely unchanged in the nutrition attention control arm with no significant difference in knowledge before or after intervention. Similarly, parents in the HPVVaxFacts intervention arm had a larger reduction in concern toward HPV vaccination compared to those in the nutrition attention control arm. However, the difference in concern scores was not significant (Table 3).

**Table 3.** Knowledge and concerns by study arm assignment (N=57).

	Knowledge, mean (SD; 95% CI)			Concerns, mean (SD; 95% CI)		
	HPVVaxFacts program	Nutrition program	*P* value	HPVVaxFacts program	Nutrition program	*P* value
Pre	2.1 (0.5; 1.2 to 3.0)	2.1 (0.4; 1.3 to 2.9)	.97	26.6 (1.8; 23.1 to 30.1)	27.3 (1.4; 24.6 to 30.1)	.75
Post	1.4 (0.3; 0.8 to 2.1)	2.2 (0.5; 1.2 to 3.1)	.22	23.2 (1.7; 19.9 to 26.5)	25.9 (1.4; 23.3 to 28.6)	.21
Post-pre	–0.6 (0.4; –1.4 to 0.1)	0.1 (0.2; –0.4 to 0.5)	.13	−3.4 (1.2; −5.8 to −1.0)	−1.4 (0.5; –2.4 to –0.4)	.14

Analysis demonstrated parents had varying degrees of intent (ie, an imperfect surrogate for vaccine hesitancy) to vaccinate their child. Before the intervention, 74% (20/27) of the parents in the HPVVaxFacts intervention arm had the intention of “Very likely” or “Somewhat likely” to get their children vaccinated compared to 60% (18/30) in the nutrition attention control arm. Post intervention, parents who reported intentions (very likely and somewhat likely) were 70% (19/27) in the HPVVaxFacts intervention arm and 63% (19/30) in the nutrition attention control arm. However, when examining pre-post changes in parents’ intentions, 3/27 (11%) parents in the HPVVaxFacts intervention arm and 1/30 (3%) parent in the nutrition attention control arm reported an increased likelihood of vaccinating their children. The pre-post intervention arm and between-arm differences were not statistically significant (Table 4).

**Table 4.** Intention by study arm.

	HPVVaxFacts (n=27)	Nutrition program (n=30)	*P* value
Preintent: HPV^a^ vaccine likelihood, n (%)			.56
Definitely not likely	4 (15)	5 (17)	
Not likely	3 (11)	7 (23)	
Somewhat likely	8 (30)	9 (30)	
Very likely	12 (44)	9 (30)	
Postintent: HPV vaccine likelihood, n (%)			.32
Definitely not likely	3 (11)	6 (20)	
Not likely	5 (19)	5 (17)	
Somewhat likely	6 (22)	11 (37)	
Very likely	13 (48)	8 (27)	
Change in intention, n (%)			.51
No change	22 (81)	27 (90)	
Less likely	2 (7)	2 (7)	
More likely	3 (11)	1 (3)	

a HPV: human papillomavirus.

**Figure 3.**
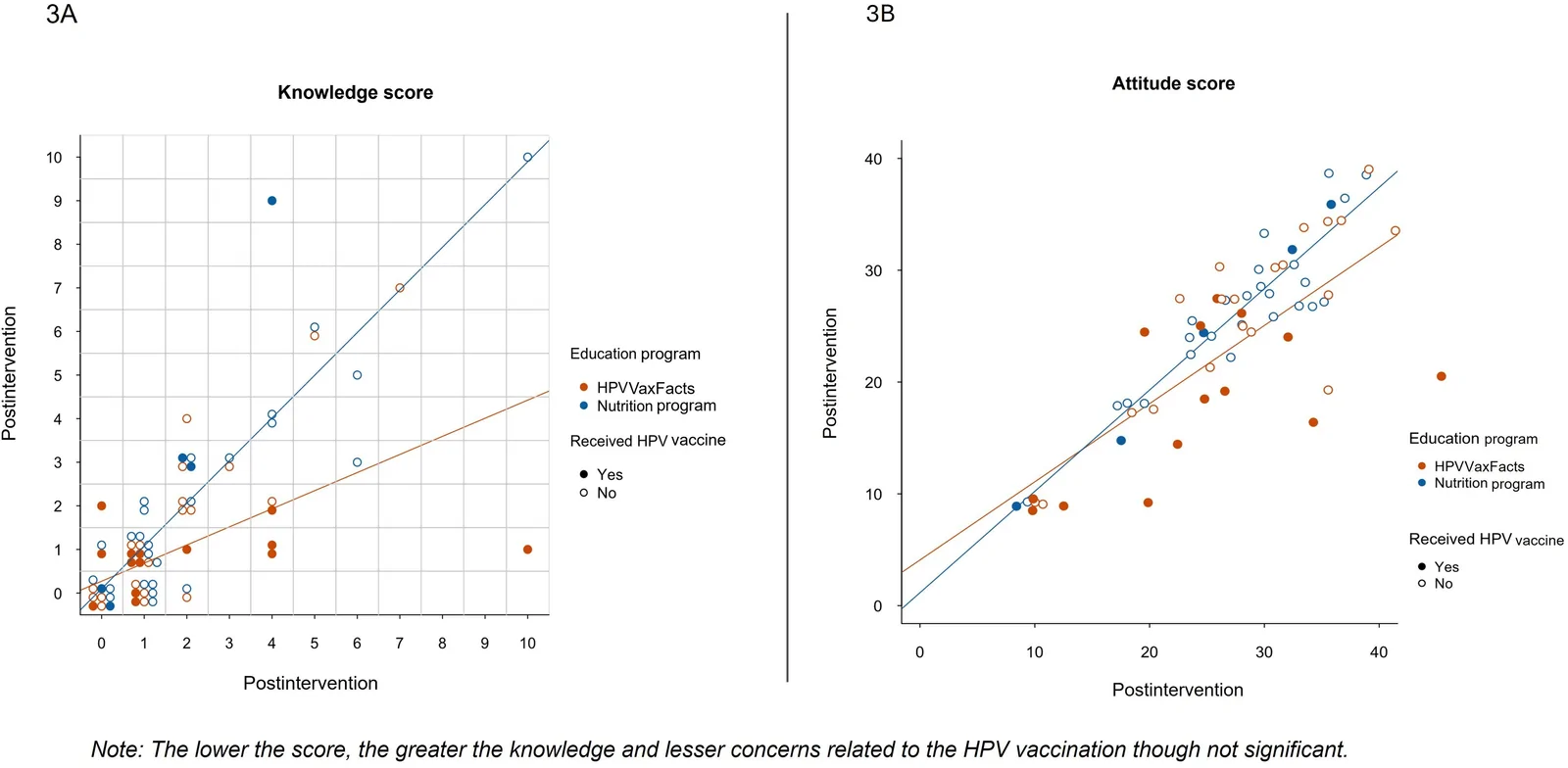
Exploratory analyses comparing pre-post scores of human papillomavirus vaccination knowledge and concerns with human papillomavirus vaccination status based on study arm assignment. HPV: human papillomavirus.

Figures 3A and 3B show an exploratory analysis between pre- and postintervention knowledge and concern scores in both study arms. For knowledge in Figure 3A, the estimated slope was 0.76 (95% CI 0.48-1.04) for HPVVaxFacts intervention arm and 0.98 (95% CI 0.78-1.18) for the nutrition attention control arm, with no evidence that the association differed by study arm (*P*_interaction_=.21). For concern in Figure 3B, the estimated slope was 0.70 (95% CI 0.52-0.89) for HPVVaxFacts intervention arm and 0.91 (95% CI 0.69-1.12) for the nutrition attention control arm, with no evidence of a differential association between study arms (*P*_interaction_=.16; Figure 3). In summary, although participants in the HPVVaxFacts intervention arm showed lower postintervention knowledge and concern scores than those in the Nutrition attention control arm, consistent with greater knowledge and fewer concerns based on the scoring of these measures, the differences between the study arms were not significant.

### Parental Top HPV Vaccination Concerns: PreSurvey and HPVVaxFacts Data Log

Only the HPVVaxFacts mobile web app allowed parents to take the quiz and confirm or change their top concerns produced via an existing algorithm. Parents in the HPVVaxFacts intervention arm were asked to identify and select their top 3 beliefs or concerns for HPV vaccination on the web app. We determined the frequency with which a concern was selected as a top 3 concern for parents. Based on the web app data, the majority of parents identified the vaccine could cause serious health problems (20/27, 74%), too many vaccines (17/27, 63%), and questioned HPV vaccine safety (12/27, 44%). Refer to Table 5 for a summary of the top 3 concerns selected for the HPVVaxFacts intervention arm participants.

**Table 5.** Top 3 selected concerns for HPVVaxFacts intervention arm participants.

Concern	WebApp (HPVVaxFacts^a^ program), n (%)
Too many vaccines could cause harm to child	17 (63)
Natural Immunity against HPV^a^ is better	7 (26)
HPV vaccine could cause serious health problems	20 (74)
Wonder if HPV vaccine is effective in preventing genital warts and cancer	8 (30)
Wonder if the HPV vaccine is safe	12 (44)
Wonder if child needs HPV vaccine to prevent cancer	6 (22)
Wonder if my child needs the HPV vaccine to prevent genital warts	1 (4)
I wonder if my child should wait until older	6 (22)
Worry that getting vaccine might make my child think it is okay to have sex	3 (11)

a HPV: human papillomavirus.

### Feasibility: Study Procedures Implementation Into Clinic Workflow

#### Data Collection and Data Availability

At Clinic Site 1, quantitative data were collected between June and September 2023. At Clinic Site 2, data collection occurred 1 week in August 2023 for back-to-school immunization week.

The data needed for study evaluation were available with less than 5% missing data from the survey, EHR for child’s age and vaccine status, and HPVVaxFacts data log tracking of parent concerns and selected top 3 HPV vaccine concerns. In total, 94% of parents completed the pre-post survey. One year post study, vaccination rates were manually pulled again by a clinic staff member to identify that 3 children in the active control group were already vaccinated when the parent enrolled in the study; these children had no record of HPV vaccination upon study enrollment, which reflects interoperability across EHRs. While these parents were excluded, it demonstrated they were interested in learning more about HPV vaccination.

#### Conceptual and Psychometric Adequacy of Measures

Psychometric measures for concerns, knowledge, and intervention acceptability were considered reliable. Cronbach α was 0.80 and higher, which is considered exceptionally good for internal consistency reliability. See measures in Methods for psychometric results.

### Parental Acceptance of HPVVaxFacts

Parents who received the HPVVaxFacts (n=27) had consistently prominent levels of acceptability for the program across all the items. Over two-thirds agreed or strongly agreed that they liked the program (mean 4.1, SD 0.7), the program met their approval (mean 4.2, SD 0.8), and it was appealing (mean 4.2, SD 0.8), and they perceived that parents should use the tool for HPV vaccine education (mean 4.2, SD 0.7). Additionally, parents perceived that HPVVaxFacts was helpful and should be used to discuss the HPV vaccine with their child (mean 4.1, SD 0.7; Table 6).

**Table 6.** Intervention acceptability by parents (n=27).

Item	Mean (SD)
Meets approval	4.2 (0.8)
Appealing	4.2 (0.8)
Like program	4.1 (0.7)
Favor of tool being used by parents	4.2 (0.7)
Helpful to discuss HPV^a^ vaccine with child	4.1 (0.7)

a HPV: human papillomavirus.

### Qualitative

A total of 19 parents completed the interviews, 9 from the HPVVaxFacts intervention arm and 10 from the nutrition attention control arm. In the HPVVaxFacts intervention arm, the mean age of the parent was 38.9 (SD 5) years. All participants were female (9/9, 100%) and non-Hispanic White (9/9, 100%). For the nutrition attention control arm, all were female (10/10, 100%), with the majority being non-Hispanic White (7/10, 70%) and almost half had an education level of GED or High School Diploma (4/10, 40%; Table S1 on parent sociodemographics in Multimedia Appendix 2).

### Themes

#### Overview

Based on participant responses, we identified 4 themes related to the study protocol. We identified one theme related to intervention acceptability using participants’ responses in the HPVVaxFacts intervention arm only. Each theme is briefly described below.

#### Theme 1: Study Protocol Acceptability and Recommendations for Improvement

##### Acceptability

Parents across both study arms perceived the study protocol as acceptable and manageable. They commented on how the clinic staff were welcoming and provided clear study enrollment information. Mobile-based delivery of each study step—consent, enrollment, presurvey, view of nutrition or education program, postsurvey—was intuitive for participants, especially those in the nutrition education arm. One nutrition program parent stated,

So, with that said, it [steps of study protocol] was fairly easy to navigate, to read through, and to get it completed, and to gain that information in a short amount of time, um, despite what I was doing.

However, receiving multiple texts for each study step posed a challenge, particularly for those in the HPVVaxFacts intervention arm. A few parents noted how clinic staff availability was helpful when questions arose. A parent in the HPVVaxFacts intervention arm stated,

The only hiccup I remember having was like, I feel like, to get from point A to point Z in the process, like it required the coordinator coming in and helping me get to where I needed to go.

Finally, parents appreciated receiving education prior to their clinic visit, which allowed them to engage with study materials without the time constraints of an in-clinic visit appointment.

Parents in the HPVVaxFacts intervention arm did not use all the mobile web app features. Three stated they did not use the videos, citing personal preference. Four did not access the kid’s corner, perceiving their child would not understand, was too young, or lacked time to view or discuss with the child. A parent in the HPVVaxFacts intervention arm stated,

Maybe when they got a little bit older, I would, but my son would, it would go right over his head. Um, but, uh, my daughter, now that she’s like 13, I hopefully would share it with her.

Parents in the nutrition attention control arm found the nutrition webpage easy to navigate and comprehensible. One parent highlighted the survey was simple, and the webpage provided immediate feedback with explanations for both correct and incorrect answers.

##### Recommendations

To optimize the study protocol, parents from both study arms identified the need to improve logistics, particularly reducing the number of text messages containing links for each step. Regarding content delivery timing, parents across study arms acknowledged their busy schedule noted the night before or day of (at home or in clinic before the visit) is an ideal time to review materials. A parent in the HPVVaxFacts intervention arm stated:

I think the day before would be good just because then they have the information to make an informed decision at the actual doctor’s visit.

However, one HPVVaxFacts parent offered a different perspective, noting an optimal window of 2 weeks before the clinic visits to get more informed about the HPV vaccine. She stated,

I would say no more than two weeks before. That’s kind of what we have found to be the sweet spot when we give deadlines for something because if you give them too much time, they’re gonna forget about it because they’re like, Oh, I’ve got plenty of time. And then if you don’t give them enough time, I mean, you know, everybody’s busy and it’s like, well, I’ve got two days and I plan I can’t get this done in two days*.*

Other suggestions related to expanding the study protocol to address all vaccines and identify alternative content delivery mechanisms. A parent in the nutrition attention control arm stated the study protocol should address all vaccines.

My honest opinion would be that every vaccine needs to be treated this way, because a lot of emphasis is given into this one vaccine…If parents are given more information, I mean if a parent wants to educate themselves and they take the initiative, they educate themselves on all the vaccines.

Another parent in the nutrition attention control arm highlighted,

I think maybe having the option of either you can get it beforehand on the phone or maybe you have it there for the provider to give to a parent to look over… I think that way you just kind of open it up to, you kind of cater to everyone that way because not everybody’s tech savvy and wants it on their phone. Some people do still like to have a piece of paper to hold and look at*.*

### Theme 2: Acceptability of and Recommendations to Improve HPVVaxFacts

#### Acceptability

Parents who participated in the HPVVaxFacts intervention arm perceived the program as user-friendly, and the layout was easy to navigate. One parent stated,

I mean, I thought the whole thing was easy to use. I mean, I texted back our primary care. They got us set up with the links and everything was very easy.

One even appreciated that the program asked targeted questions to narrow parental concerns and delivered tailored content. Parents found the content comprehensible and accessible, particularly on the mobile phone.

I thought it was good information. I liked that it was easily accessible and pretty quick to read through and understand.

Another parent noted*,*

*I think everything was pretty, pretty easy to understand and follow.*

However, program completion was unclear for 2 parents. One parent stated,

A little unclear, like, I even messaged him and I was like, I think I finished everything, but I'm not sure that I did. And they were like, we think you did too, but we're not sure either.

#### Recommendations

Parents suggested improving the web app and adding parent testimonials to demonstrate how others made vaccination decisions. One parent stated,

I think it’s always good, especially for people who are, who have reservations to see. Stories of others who say, Hey, you know, I was really on the fence about this because I thought this, but, you know, I got this information and it may be understand that that wasn’t the case because of whatever*.*

Parents also requested more detailed information on “extreme” side effects and misinformation. One parent preferred links to research articles over the videos that discussed each concern.

Two parents highlighted the need for the app to be multilingual for greater reach. Parents wanted a reminder via text or email from providers to use the mobile web app to help prepare questions in advance. A few parents suggested increasing mobile web app availability for use by health care agencies, schools, and provider-led public service announcements (PSAs) to extend reach to parents. A parent stated*,*

I mean, my initial reaction is things like schools, but I know you have to be so careful with what kind of stuff you put out in schools nowadays*.*

Another parent recommended,

If it became if it was, like, adopted by the state, the health department would be a really good channel, like, maybe the website through the health department.

Two parents recommended that this program should be applied across all vaccines for parents to understand the benefits of vaccination. Specifically, the parent stated,

I think that if they had it for more vaccines that people would have a better understanding as to what it is that they are subjecting their child to by having the vaccinations, the benefits*.*

### Theme 3: Perceived Benefits of HPVVaxFacts

Parents described 4 benefits from participating in the HPVVaxFacts intervention arm. We summarize these benefits below.

#### Confirmed or Improved Knowledge for Decision-Making

Parents commonly reported that HPVVaxFacts improved or reaffirmed their knowledge of HPV and the vaccine, regardless of their baseline knowledge level. One parent stated,

It reaffirmed kind of what I already knew, and it did give me additional information that I might not necessarily have had or even have thought to ask the doctor about*.*

Another parent described,

I thought it was very informative. A lot of the stuff I already knew, but I could see how, especially with new parents or parents who aren’t as hypervigilant, could benefit from having that information if they didn’t already know it*.*

Some parents felt better equipped to make an informed decision after reviewing the program. A parent stated,

I feel like the information was clear and straightforward, and I think it was presented in a way to help parents more understand the vaccine and make an informed decision*.*

#### Preclinic Information Improves Appointment Efficiency

All parents valued receiving HPV vaccination information preclinic visit and found it feasible. The majority of parents had adequate time to review the information and form a decision. A parent stated,

I liked having it ahead of time because then by the time I got in there, I had all the information that I needed in order to make the decision on whether or not we were going to do it*.*

Several parents noted that information prior to the clinic visit allowed appointments to be more efficient with fewer questions. One parent emphasized,

I think that with the program, it gave me all the information that was needed to be able to just make the appointment smoother and just be able to like go in there and have our doctor’s appointment and not have to worry so much about any of the questions. Because I felt like it was there for me to be able to know all the things before, I got there*.*

Other parents noted HPVVaxFacts helped facilitate the discussion with the child’s provider. A parent stated,

Honestly, it makes the appointment go faster. I mean, I know that the doctors are busy, so it’s good to just have before so you just know what, what exactly it is before you, you know, you end up doing it if you do decide to do it or whatever*.*

However, 2 parents wreported no provider recommendation or discussion on the HPV vaccination during the clinic visit, making previsit information particularly helpful in their decision-making.

#### Trust in Provider-Delivered Information

All parents appreciated that the program was delivered by their child’s provider, who was deemed a trusted information source. A parent stated,

Yeah, I think it [HPVVaxFacts] was provided by his pediatrician. That’s probably the best thing about it. In my opinion.

Parents acknowledged widespread misinformation on vaccines and identified how this app could address those concerns. One parent stated,

Yeah, I definitely trust information provided through his pediatrician more so than just something you find online because I feel like it’s easy to find supporting information for your bias ideas online. I can find something from the pediatrician. I’m going to trust that further than whatever I research on the Internet*.*

Furthermore, they valued the credibility of provider-sourced information. One parent lauded,

There is way too much out there, and you don't know what you can trust and what you can't trust. Um, so definitely coming from a clinic and a trusted place is very comforting*.*

#### Facilitates Parent-Child Discussion on HPV Vaccine

One parent used the kid corner to guide discussion with her child, while another found the information “lay” enough to discuss the HPV vaccine with her child. A parent shared,

I remember, thinking that it had good information for me to be able to explain it to my children in a way that they would understand better*.*

Parents valued the program to facilitate parent-child conversations on vaccines such as HPV, with some preferring to use their older children who could benefit from these open discussions. A parent stated,

I think for older children, especially and where they are having conversations that are more open with their children about vaccines*.*

### Theme 4: Perceived Barriers to HPVVaxFacts Use

Most parents reported no barriers to using the program. A parent stated,

“*I don't think so. I don't recall having anything that was, that I would consider a barrier.*”

Three parents highlighted potential barriers that were not program-specific. One barrier was that some parents may lack access to the mobile web app. Another barrier older or not technologically “savvy” parents may have difficulty with web app navigation. A parent stated,

That [navigating a technology] would be a little bit more difficult, like later 50s, because I know I’m I had my daughter when I was 22, but I know a lot of parents aren’t having them until like they’re in their, you know, early to late 30s*.*

Finally, one parent noted that some parents may not trust their health care provider and the research team or the information provided. It was further recommended to use alternative distribution channels (eg, media, social media, PSA) to reach these individuals.

### Clinic Staff Consultation: Provider Feedback

Overall, providers perceived the program as beneficial for their clinic. Particularly, the top benefit was offering preclinic education visits to their patients. This complemented their recommendations and lessened clinic time for education. Another perceived benefit was the increased vaccination rates in the clinic because of the intervention. Regarding barriers, providers and clinic staff highlighted the need to improve logistics for large-scale implementation. Because the intervention was manual and required efforts from clinic staff and the research team to aid in recruitment and retention, providers questioned the feasibility of recruitment and retention procedures but still perceived offering preclinic information in general as feasible. However, providers and staff stated they understood this was a pilot study and hoped their suggestions improved future implementation.

## Discussion

### Principal Findings

Our study assessed feasibility, acceptability, and preliminary efficacy of the theory-informed, mobile web app HPVVaxFacts designed to decrease concerns and increase knowledge, intentions, and uptake of the vaccine among children aged 9 to 17 years. This study is one of the first to use a multimethod evaluation to assess mHealth interventions to increase HPV vaccination. More importantly, HPVVaxFacts is one of the first mobile web apps to identify and tailor information to parental concerns while supporting autonomy in both parental decision-making and educating their child using an optional kid’s corner on the web app.

### Feasibility

Our study reported feasibility across multiple metrics. The enrollment rate was 84%, and the random allocation achieved 100% recruitment. The retention rates in both study arms were 75% in the HPV program and 94% in the nutrition program. Notably, approximately 4 out of 5 (81%) parents in the intervention arm viewed HPVVaxFacts. These rates are considered good, especially with the sensitive context surrounding HPV vaccination and typical survey response rates. The engagement of clinic staff and parents in the development of the study protocol, a known factor in improving study outcomes (eg, logistics and behavior) [51], partly attributes to this success.

Additionally, the Cronbach α for concerns, knowledge, and intervention acceptability scales were considered reliable, further supporting study feasibility and potential deployment of these scales in a larger study. However, because one clinic had vaccination rates substantially increase prior to study implementation, this underscores the importance of conducting a feasibility assessment at multiple checkpoints before and during study implementation. Furthermore, both providers and participants highlighted the logistical barrier of study steps being manual, a factor which may have affected response rates at different steps in the research process. This supports the need to automate these steps for future efficacy trials to improve recruitment and retention.

Interestingly, parents identified factors that could influence use of HPVVaxFacts long-term in research and in clinical practice. For protocol application, improving the study logistics was commonly mentioned by both parents and providers, though not reflective of app functionality. This suggests that the tool alone could be applied in real-world settings with minimal human involvement, a major advantage for sustainability. Similar to past studies [52], parents identified limited access to technology (eg, mobile web app) as a potential barrier reflecting the “digital divide” in America [53]. Some parents, particularly older parents or non–English-speaking parents, may struggle to navigate the mobile web app, which has been identified as a barrier previously [54]. Some parents may mistrust a provider who delivered resources because of broader mistrust in health care. This demonstrates how mistrust can extend beyond the individual to tangibles perceived to be associated with the individual [55]. These findings suggest that feasibility of HPVVaxFacts can be improved using multilingual languages and addressing the digital divide (eg, physical infrastructure expansion, training, and tech support). Also, alternative strategies for providing health care information should be considered, including multiple dissemination channels and provider-led initiatives to build support and trust.

### Acceptability

Acceptability was considered good with a rate of 74% or higher with room for improvement. Of interest, acceptability of HPVVaxFacts centered around the ability of the app to tailor to the parents’ needs. Particularly, parents were able to access the app when available (previsit information), identify their top vaccine concerns, and view the modality that best served their need to get their concerns addressed. This suggests the importance of a multilevel, tailored approach that is cost-effective to target parent decision-making processes for vaccination, creating a unique opportunity for this tool to have broad reach. Other acceptable components of the mobile web app, though deemed unsurprising, were its comprehensible content, ability to navigate, and being time-friendly for use. Previsit information has been supported by past studies [26], but its use in the context of a mobile app has been underexplored. Parents lauded it for reasons such as lessened time in clinic or improved preparation for the visit, an acceptable strategy we add to the literature. Our findings also support that increased acceptability of mobile web apps for parent-child discussion could be achieved via provision of parental autonomy to share select information or allow parents to use information to guide a discussion with the child without direct access. This should be further explored as mHealth continues to evolve to expand the reach of health information for caregivers and/or their children.

Not only did parents view HPVVaxFacts to improve feasibility through content being multilingual, but it was also viewed to increase acceptability. Particularly, the suggestion to expand this app content to different languages and its use in alternative settings that provide vaccination to increase acceptability reflects the altruism of these parents. Walker et al [56] found that parents with higher interpersonal altruism had higher odds of agreeing with HPV-specific state vaccine requirements, a confirmation of our qualitative findings. These findings suggest the need for inclusivity when developing strategies and tools that promote HPV vaccination among children. Exploring the app’s use in alternative immunization settings supports the HPV National Roundtables’ goal to increase HPV vaccination in these settings.

### Preliminary Efficacy

Similar to other mHealth studies [26], HPVVaxFacts increased HPV vaccine initiation rates among children at significantly higher rates compared to the nutrition program. Interestingly, this increase occurred despite no significant differences in knowledge, concerns, or intentions between the study arms. Because knowledge and attitudes had a positive trajectory through the increase in effect size in the intervention arm compared to the control arm, this could reflect the pilot study being underpowered for these secondary outcomes. This is a common outcome, as many pilot studies are powered for testing feasibility and not proving an intervention’s efficacy [57]. Also, it remains unclear whether, and to what extent, this effect was mediated through provider engagement or indirect engagement via app delivery through the EHR. Nevertheless, our qualitative findings support that parental willingness to get vaccinated was due to receipt of the information from the provider. Because parents’ most trusted information source [55,58] is providers and their recommendation is critical for uptake [59], future studies should explore in-depth whether and how provider communication modalities can be leveraged to complement provider recommendations and communication in-clinic with a goal to increase HPV vaccination compliance. Finally, these findings could also reflect a ceiling effect in which a significant portion of participants in both the HPV and nutrition programs already had positive intentions to vaccinate [60]. Future studies could either recruit parents who are “not likely” or “somewhat likely” only or use baseline status to stratify analysis, both strategies to better capture intervention effects [61]. Finally, it was not surprising that the top 3 concerns identified by these parents centered around vaccine safety, the leading barrier to vaccine hesitancy and suboptimal rates [62].

### Limitations

First, this study was conducted in 2 pediatric urban clinics in Tennessee in which the primary clinic for recruitment was predominantly female and non-Hispanic White. Collectively, this may limit generalizability to other populations, including those living in different geographical regions and settings along with racial and ethnic groups. Second, selection bias may have occurred if some parents with low mobile app literacy or who did not have a mobile app did not participate, which should be explored in future studies. However, we have begun to take steps to improve inclusion through the provision of HPVVaxFacts in Spanish. Third, this study had a small sample size, which limited our ability to detect a difference for some of the study outcomes (eg, concerns and intention) and whether those outcomes are a result of clinic demographics and protocol. Also, the small number of parents recruited at Clinic Site 2 made it impossible to assess whether clinic-level differences influenced results. Fourth, there were updates on the app during the feasibility study; however, study implementation metrics, including recruitment and retention, improved over time. Fifth, because parents agreed to be in the study and the invite mentioned HPV vaccination, parents may be more engaged or interested in receiving information. This should be explored in a larger study.

Sixth, some parents were lost due to their child already being vaccinated during study participation and study logistics, in which we could not recruit additional participants due to the study duration ending at both clinics. However, this reflects EHR interoperability, a commonly cited issue confirmed by clinic staff [63]. Seventh, we only studied the impact of HPVVaxFacts on initiation rates. The impact on completion rates (ie, 2 doses of HPV vaccine among children younger than 15 years old and 3 doses among those older than 15 years old) [64] will be explored in a future study, as we are exploring reasons for noncompliance and informational needs of parents to inform the update of messaging on the mobile web app to be more comprehensive. Eighth, for the interviews, there may be potential for recall bias, which increases due to the variable time between parent participation and the interview due to demanding study logistics. Ninth, some mobile web app components (ie, average session length) could not be tracked due to financial constraints. However, it is currently being updated to address this limitation. Tenth, small discrepancy between the HPVVaxFacts website content and knowledge scale could have led to a lack of significant improvement in knowledge. We were also unable to track participants’ engagement within the website that may have varied from full review to partial or no interaction with the content. It is possible that the nutrition education within the attention control arm could have influenced parents to accept and/or obtain the first dose of the HPV vaccination for their child. Finally, the nutrition attention control arm did not select their top concerns as this was not an option on the app. Therefore, we are unable to compare parental top concerns by study arm.

### Conclusion

Our pilot study suggests HPVVaxFacts is feasible and acceptable to provide tailored information to parents’ preclinic visits to aid parent decision-making for HPV vaccination and ultimately increase uptake. This study’s outcomes, both unclear yet offer a positive trajectory, lay the foundation for a full RCT to test the efficacy of HPVVaxFacts being offered preclinic visit to increase HPV vaccine initiation and completion rates. Future studies can explore the mediating (eg, provider recommendation and communication) and moderating factors for successful intervention effects with a large sample size. Findings also reinforce that provider modalities such as mHealth are acceptable and can complement provider communication to address parental HPV vaccine concerns.

## Supplementary material

10.2196/78910 Multimedia Appendix 1 HPVVaxFacts pilot, feasibility trial CONSORT.

10.2196/78910 Multimedia Appendix 2 Qualitative interviewee sociodemographics (n=19).

10.2196/78910 Checklist 1 CONSORT-eHEALTH checklist (V 1.6.1).

## Abbreviations

CONSORT: Consolidated Standards of Reporting Trials
CONSORT-EHEALTH: Consolidated Standards of Reporting Trials of Electronic and Mobile Health Applications and Online Telehealth
EBP: evidence-based practice
EHR: electronic health record
GED: General Educational Development
HIPAA: Health Insurance Portability and Accountability Act
HPV: human papilloma virus
IRB: Institutional Review Board
mHealth: mobile health
PSA: public service announcement
RCT: randomized control trial
