# Supplementary material for: A Multimethod Evaluation to Assess Feasibility, Acceptability, and Preliminary Efficacy of HPVVaxFacts, a Tailored Mobile Web App, for Parents With Unvaccinated Children: Pilot 2-Arm Randomized Controlled Trial
Source: JMIR Cancer. 2026 Sep 9;12:e78910. doi: 10.2196/78910 (PMC13557614; doi:10.2196/78910)
Supplement: Supplementary file 1 [file cancer-v12-e78910-s003.pdf]

CONSORT-EHEALTH (V 1.6.1) - Submission/Publication Form

The CONSORT-EHEALTH checklist is intended for authors of randomized trials evaluating web-based and Internet-based applications/interventions, including mobile interventions, electronic games (incl multiplayer games), social media, certain telehealth applications, and other interactive and/or networked electronic applications. Some of the items (e.g. all subitems under item 5 - description of the intervention) may also be applicable for other study designs.

The goal of the CONSORT EHEALTH checklist and guideline is to be
a) a guide for reporting for authors of RCTs,
b) to form a basis for appraisal of an ehealth trial (in terms of validity)

CONSORT-EHEALTH items/subitems are MANDATORY reporting items for studies published in the Journal of Medical Internet Research and other journals / scientific societies endorsing the checklist.

Items numbered 1., 2., 3., 4a., 4b etc are original CONSORT or CONSORT-NPT (non-pharmacologic treatment) items.
Items with Roman numerals (i., ii, iii, iv etc.) are CONSORT-EHEALTH extensions/clarifications.

As the CONSORT-EHEALTH checklist is still considered in a formative stage, we would ask that you also RATE ON A SCALE OF 1-5 how important/useful you feel each item is FOR THE PURPOSE OF THE CHECKLIST and reporting guideline (optional).

Mandatory reporting items are marked with a red *.
In the textboxes, either copy & paste the relevant sections from your manuscript into this form - please include any quotes from your manuscript in QUOTATION MARKS,
or answer directly by providing additional information not in the manuscript, or elaborating on why the item was not relevant for this study.

YOUR ANSWERS WILL BE PUBLISHED AS A SUPPLEMENTARY FILE TO YOUR PUBLICATION IN JMIR AND ARE CONSIDERED PART OF YOUR PUBLICATION (IF ACCEPTED).
Please fill in these questions diligently. Information will not be copyedited, so please use proper spelling and grammar, use correct capitalization, and avoid abbreviations.

DO NOT FORGET TO SAVE AS PDF _AND_ CLICK THE SUBMIT BUTTON SO YOUR ANSWERS ARE IN OUR DATABASE !!!

Citation Suggestion (if you append the pdf as Appendix we suggest to cite this paper in the caption):
Eysenbach G, CONSORT-EHEALTH Group

CONSORT-EHEALTH: Improving and Standardizing Evaluation Reports of Web-based and Mobile Health Interventions
J Med Internet Res 2011;13(4):e126
URL: http://www.jmir.org/2011/4/e126/
doi: 10.2196/jmir.1923
PMID: 22209829

docjlc1126@gmail.com Switch account

Not shared

Draft saved

* Indicates required question

Your name *

First Last

Jennifer Cunningham-Erves

Primary Affiliation (short), City, Country *

University of Toronto, Toronto, Canada

Vanderbilt University Medical Center, Nashville

Your e-mail address *

abc@gmail.com

jennifer.erves@vumc.org

Title of your manuscript *

Provide the (draft) title of your manuscript.

A multi-method evaluation to assess feasibility, acceptability, and preliminary efficacy of HPVVaxFacts, a tailored mobile web app, for parents with unvaccinated children: A pilot 2-arm randomized control trial

Name of your App/Software/Intervention *

If there is a short and a long/alternate name, write the short name first and add the long name in brackets.

HPVVaxFacts

Evaluated Version (if any)

e.g. "V1", "Release 2017-03-01", "Version 2.0.27913"

Not applicable.

Language(s) *

What language is the intervention/app in? If multiple languages are available, separate by comma (e.g. "English, French")

English, Spanish

URL of your Intervention Website or App

e.g. a direct link to the mobile app on app in appstore (itunes, Google Play), or URL of the website. If the intervention is a DVD or hardware, you can also link to an Amazon page.

https://www.hpvvaxfacts.com/

URL of an image/screenshot (optional)

Your answer

Accessibility *

Can an enduser access the intervention presently?

- ○ access is free and open
- ○ access only for special usergroups, not open
- ○ access is open to everyone, but requires payment/subscription/in-app purchases
- ○ app/intervention no longer accessible
- ◉ Other: App is closed for future development but homepage may be accessec

Primary Medical Indication/Disease/Condition *

e.g. "Stress", "Diabetes", or define the target group in brackets after the condition, e.g. "Autism (Parents of children with)", "Alzheimers (Informal Caregivers of)"

HPV and associated cancers

Primary Outcomes measured in trial *

comma-separated list of primary outcomes reported in the trial

HPV vaccination initiation

Secondary/other outcomes

Are there any other outcomes the intervention is expected to affect?

knowledge, attitudes, intentions, acceptability, feasibility

Recommended "Dose" *

What do the instructions for users say on how often the app should be used?

- ( ) Approximately Daily
- ( ) Approximately Weekly
- ( ) Approximately Monthly
- ( ) Approximately Yearly
- (•) "as needed"
- ( ) Other:

Approx. Percentage of Users (starters) still using the app as recommended after 3 months *

- (selected) unknown / not evaluated
- 0-10%
- 11-20%
- 21-30%
- 31-40%
- 41-50%
- 51-60%
- 61-70%
- 71%-80%
- 81-90%
- 91-100%
- Other:

Overall, was the app/intervention effective? *

- (selected) yes: all primary outcomes were significantly better in intervention group vs control
- partly: SOME primary outcomes were significantly better in intervention group vs control
- no statistically significant difference between control and intervention
- potentially harmful: control was significantly better than intervention in one or more outcomes
- inconclusive: more research is needed
- Other:

Article Preparation Status/Stage *

At which stage in your article preparation are you currently (at the time you fill in this form)

- ( ) not submitted yet - in early draft status
- ( ) not submitted yet - in late draft status, just before submission
- ( ) submitted to a journal but not reviewed yet
- (•) submitted to a journal and after receiving initial reviewer comments
- ( ) submitted to a journal and accepted, but not published yet
- ( ) published
- ( ) Other:

Journal *

If you already know where you will submit this paper (or if it is already submitted), please provide the journal name (if it is not JMIR, provide the journal name under "other")

- ( ) not submitted yet / unclear where I will submit this
- ( ) Journal of Medical Internet Research (JMIR)
- ( ) JMIR mHealth and UHealth
- ( ) JMIR Serious Games
- ( ) JMIR Mental Health
- ( ) JMIR Public Health
- ( ) JMIR Formative Research
- ( ) Other JMIR sister journal
- (•) Other: JMIR Cancer

Is this a full powered effectiveness trial or a pilot/feasibility trial? *

- (selected) Pilot/feasibility
- ( ) Fully powered

Manuscript tracking number *

If this is a JMIR submission, please provide the manuscript tracking number under "other" (The ms tracking number can be found in the submission acknowledgement email, or when you login as author in JMIR. If the paper is already published in JMIR, then the ms tracking number is the four-digit number at the end of the DOI, to be found at the bottom of each published article in JMIR)

- ( ) no ms number (yet) / not (yet) submitted to / published in JMIR
- (selected) Other: #78910

TITLE AND ABSTRACT

1a) TITLE: Identification as a randomized trial in the title

1a) Does your paper address CONSORT item 1a? *

I.e does the title contain the phrase "Randomized Controlled Trial"? (if not, explain the reason under "other")

- (selected) yes
- ( ) Other:

1a-i) Identify the mode of delivery in the title

Identify the mode of delivery. Preferably use “web-based” and/or “mobile” and/or “electronic game” in the title. Avoid ambiguous terms like “online”, “virtual”, “interactive”. Use “Internet-based” only if Intervention includes non-web-based Internet components (e.g. email), use “computer-based” or “electronic” only if offline products are used. Use “virtual” only in the context of “virtual reality” (3-D worlds). Use “online” only in the context of “online support groups”. Complement or substitute product names with broader terms for the class of products (such as “mobile” or “smart phone” instead of “iphone”), especially if the application runs on different platforms.

	1	2	3	4	5	
subitem not at all important	○	○	○	○	◉	essential

Clear selection

Does your paper address subitem 1a-i? *

Copy and paste relevant sections from manuscript title (include quotes in quotation marks "like this" to indicate direct quotes from your manuscript), or elaborate on this item by providing additional information not in the ms, or briefly explain why the item is not applicable/relevant for your study

"A multi-method evaluation to assess feasibility, acceptability, and preliminary efficacy of HPVVaxFacts, a tailored mobile web app, for parents with unvaccinated children: A pilot 2-arm randomized control trial"

1a-ii) Non-web-based components or important co-interventions in title

Mention non-web-based components or important co-interventions in title, if any (e.g., “with telephone support”).

	1	2	3	4	5	
subitem not at all important	◉	○	○	○	○	essential

Clear selection

Does your paper address subitem 1a-ii?

Copy and paste relevant sections from manuscript title (include quotes in quotation marks "like this" to indicate direct quotes from your manuscript), or elaborate on this item by providing additional information not in the ms, or briefly explain why the item is not applicable/relevant for your study

Not applicable for our study as there were no co-interventions or non-web based components.

1a-iii) Primary condition or target group in the title

Mention primary condition or target group in the title, if any (e.g., “for children with Type I Diabetes”) Example: A Web-based and Mobile Intervention with Telephone Support for Children with Type I Diabetes: Randomized Controlled Trial

	1	2	3	4	5	
subitem not at all important	○	○	○	○	◉	essential

Clear selection

Does your paper address subitem 1a-iii? *

Copy and paste relevant sections from manuscript title (include quotes in quotation marks "like this" to indicate direct quotes from your manuscript), or elaborate on this item by providing additional information not in the ms, or briefly explain why the item is not applicable/relevant for your study

"for parents with unvaccinated children"

1b) ABSTRACT: Structured summary of trial design, methods, results, and conclusions

NPT extension: Description of experimental treatment, comparator, care providers, centers, and blinding status.

1b-i) Key features/functionalities/components of the intervention and comparator in the METHODS section of the ABSTRACT

Mention key features/functionalities/components of the intervention and comparator in the abstract. If possible, also mention theories and principles used for designing the site. Keep in mind the needs of systematic reviewers and indexers by including important synonyms. (Note: Only report in the abstract what the main paper is reporting. If this information is missing from the main body of text, consider adding it)

	1	2	3	4	5	
subitem not at all important	○	○	○	○	◉	essential

Clear selection

Does your paper address subitem 1b-i? *

Copy and paste relevant sections from the manuscript abstract (include quotes in quotation marks "like this" to indicate direct quotes from your manuscript), or elaborate on this item by providing additional information not in the ms, or briefly explain why the item is not applicable/relevant for your study

"HPVVaxFacts is a novel, mobile web app delivered to a parent's phone before clinic visits, offering education tailored to their concerns based on the Health Belief Model and Theory of Planned Behavior."

"We conducted a pilot, 2-arm randomized control trial comparing two groups: tailored education via HPVVaxFacts (n=27) (intervention) compared to nutrition education via a mobile web app offering children's nutrition information (n=30) (attention control) in two urban clinics in Tennessee from June to September 2023."

1b-ii) Level of human involvement in the METHODS section of the ABSTRACT

Clarify the level of human involvement in the abstract, e.g., use phrases like “fully automated” vs. “therapist/nurse/care provider/physician-assisted” (mention number and expertise of providers involved, if any). (Note: Only report in the abstract what the main paper is reporting. If this information is missing from the main body of text, consider adding it)

	1	2	3	4	5	
subitem not at all important	◉	○	○	○	○	essential

Clear selection

Does your paper address subitem 1b-ii?

Copy and paste relevant sections from the manuscript abstract (include quotes in quotation marks "like this" to indicate direct quotes from your manuscript), or elaborate on this item by providing additional information not in the ms, or briefly explain why the item is not applicable/relevant for your study

This is not the main context of the manuscript but we do discuss in the manuscript.

1b-iii) Open vs. closed, web-based (self-assessment) vs. face-to-face assessments in the METHODS section of the ABSTRACT

Mention how participants were recruited (online vs. offline), e.g., from an open access website or from a clinic or a closed online user group (closed usergroup trial), and clarify if this was a purely web-based trial, or there were face-to-face components (as part of the intervention or for assessment). Clearly say if outcomes were self-assessed through questionnaires (as common in web-based trials). Note: In traditional offline trials, an open trial (open-label trial) is a type of clinical trial in which both the researchers and participants know which treatment is being administered. To avoid confusion, use "blinded" or "unblinded" to indicated the level of blinding instead of "open", as "open" in web-based trials usually refers to "open access" (i.e. participants can self-enrol). (Note: Only report in the abstract what the main paper is reporting. If this information is missing from the main body of text, consider adding it)

	1	2	3	4	5	
subitem not at all important	○	○	○	○	◉	essential

Clear selection

Does your paper address subitem 1b-iii?

Copy and paste relevant sections from the manuscript abstract (include quotes in quotation marks "like this" to indicate direct quotes from your manuscript), or elaborate on this item by providing additional information not in the ms, or briefly explain why the item is not applicable/relevant for your study

"Participants were recruited using patient intake software- Phreesia and eClinicalWorks."

"Parents were assessed at baseline and immediately post-education program via surveys and follow-up interviews, along with a provider consultation. Vaccination rates were assessed at 12 months post-intervention. Staff consultation was 1-month post-intervention, and parent interviews up to 9 months post-intervention."

1b-iv) RESULTS section in abstract must contain use data

Report number of participants enrolled/assessed in each group, the use/uptake of the intervention (e.g., attrition/adherence metrics, use over time, number of logins etc.), in addition to primary/secondary outcomes. (Note: Only report in the abstract what the main paper is reporting. If this information is missing from the main body of text, consider adding it)

	1	2	3	4	5	
subitem not at all important	○	○	○	○	◉	essential

Clear selection

Does your paper address subitem 1b-iv?

Copy and paste relevant sections from the manuscript abstract (include quotes in quotation marks "like this" to indicate direct quotes from your manuscript), or elaborate on this item by providing additional information not in the ms, or briefly explain why the item is not applicable/relevant for your study

"Results: We recruited participants over 4 months. With no differences across socio-demographics, most parents were female (91%), White (77%), had ≤ $80,000 household income (56%), and had some college or less (48%). Following the HPVVaxFacts intervention, 48% of unvaccinated children initiated vaccination compared to 17% in the nutrition arm (Difference 0.24, 95% CI (0.033, 0.46); p=0.011). The HPVVaxFacts arm showed a larger reduction in mean concern level (3.4) compared to the nutrition arm (1.4; Difference 2.0, 95% CI (-0.70, 4.6) though not statistically significant. Eighty percent of parents viewed the HPVVaxFacts web app. Majority of parents who received HPVVaxFacts stated it met their approval (82%). Qualitative themes were: 1) Study Protocol Acceptability and Recommendations for Improvement; 2) Acceptability of and Recommendations to Improve HPVVaxFacts; 3) Perceived Benefits of HPVVaxFacts; and 4) Perceived Barriers to using HPVVaxFacts."

1b-v) CONCLUSIONS/DISCUSSION in abstract for negative trials

Conclusions/Discussions in abstract for negative trials: Discuss the primary outcome - if the trial is negative (primary outcome not changed), and the intervention was not used, discuss whether negative results are attributable to lack of uptake and discuss reasons. (Note: Only report in the abstract what the main paper is reporting. If this information is missing from the main body of text, consider adding it)

	1	2	3	4	5	
subitem not at all important	○	○	○	○	◉	essential

Clear selection

Does your paper address subitem 1b-v?

Copy and paste relevant sections from the manuscript abstract (include quotes in quotation marks "like this" to indicate direct quotes from your manuscript), or elaborate on this item by providing additional information not in the ms, or briefly explain why the item is not applicable/relevant for your study

"Conclusion: HPV vaccination initiation rates were significantly greater in the intervention arm compared to the attention control arm. Furthermore, the mobile web app was feasible, and parents found this mobile web app acceptable. Next steps include conducting a well-powered efficacy trial to determine the impact of HPVVaxFacts on HPV initiation vaccine rates and parental hesitancy factors, as well as to explore an interaction, effect modification, and mediation among different variables."

INTRODUCTION

2a) In INTRODUCTION: Scientific background and explanation of rationale

2a-i) Problem and the type of system/solution

Describe the problem and the type of system/solution that is object of the study: intended as stand-alone intervention vs. incorporated in broader health care program? Intended for a particular patient population? Goals of the intervention, e.g., being more cost-effective to other interventions, replace or complement other solutions? (Note: Details about the intervention are provided in "Methods" under 5)

	1	2	3	4	5	
subitem not at all important	○	○	○	○	◉	essential

Clear selection

Does your paper address subitem 2a-i? *

Copy and paste relevant sections from the manuscript (include quotes in quotation marks "like this" to indicate direct quotes from your manuscript), or elaborate on this item by providing additional information not in the ms, or briefly explain why the item is not applicable/relevant for your study

"Parental vaccine hesitancy remains a critical barrier to suboptimal rates of HPV vaccination. Studies show up to 63% of US parents are hesitant [1,2] (i.e. reluctant or refuse to vaccinate) despite its availability and proven effectiveness [3]. This contributes to stalled HPV vaccination rates among adolescents aged 13-17 years, 63% in the US and 64% in Tennessee in 2022 [4]. Unvaccinated adolescents are at risk for HPV infection, which is implicated in almost all cervical cancers and 63-90% of penile, vulvar, vaginal, anal, and oropharyngeal cancers [5]. with many have rising incidence rates [6]. Based on several studies among US parents, predictors for HPV vaccine hesitancy are complex and multifactorial [7–9]. Common reasons include concern of safety/side effects, lack of knowledge on HPV and associated cancers, and lack of or poor-quality provider recommendations and communication [10]. Hesitant parents are often dissatisfied with patient–provider communication, further undermining their confidence and decision-making on HPV vaccine initiation and completion [11–14]. There is an urgent call to identify evidence-based practices (EBPs) to address vaccine hesitancy globally and nationally [4,13,15]."

2a-ii) Scientific background, rationale: What is known about the (type of) system

Scientific background, rationale: What is known about the (type of) system that is the object of the study (be sure to discuss the use of similar systems for other conditions/diagnoses, if appropiate), motivation for the study, i.e. what are the reasons for and what is the context for this specific study, from which stakeholder viewpoint is the study performed, potential impact of findings [2]. Briefly justify the choice of the comparator.

	1	2	3	4	5	
subitem not at all important	○	○	○	○	◉	essential

Clear selection

Does your paper address subitem 2a-ii? *

Copy and paste relevant sections from the manuscript (include quotes in quotation marks "like this" to indicate direct quotes from your manuscript), or elaborate on this item by providing additional information not in the ms, or briefly explain why the item is not applicable/relevant for your study

"Few interventions to date have been found impactful in addressing parental HPV vaccine hesitancy. Mobile health (mHealth) interventions show promise in enhancing traditional medical care. With provider-based strategies (e.g., a strong recommendation, motivational interviewing), mHealth intervention could positively impact parental concerns by countering misinformation or address lack of information about HPV vaccination [16–22]. Some data exist on mHealth in promoting HPV vaccination, but important limitations remain. A systematic review from January 2017 to July 2022 identified 24 digital health interventions for HPV vaccination with only four employing theory-based mHealth interventions [23–26]. Limitations of these interventions are: 1) a focus on general HPV vaccine promotion and not unique concerns of vaccine-hesitant parents; 2) targeted only girls or younger adolescents; and 3) limited testing across diverse socio-demographics and geography. Two of these studies tailored information through the use of reminders or by individual vaccine status [23,26–29]. There remains an unmet need to develop an efficacious mHealth program that provides parents succinct, resonating messages individually tailored to their concerns on the HPV vaccine for use by providers to supplement parent-provider in-clinic discussion [30,31]."

2b) In INTRODUCTION: Specific objectives or hypotheses

Does your paper address CONSORT subitem 2b? *

Copy and paste relevant sections from the manuscript (include quotes in quotation marks "like this" to indicate direct quotes from your manuscript), or elaborate on this item by providing additional information not in the ms, or briefly explain why the item is not applicable/relevant for your study

"This study's objective was to conduct a pilot test of a mobile web app intervention, HPVVaxFacts, designed to provide tailored, pre-visit education to improve HPV vaccination rates. We hypothesized that: The HPVVaxFacts intervention group will have acceptability and feasibility rates at ≥70%; 2) Parents in the intervention arm will demonstrate greater increases in knowledge, attitudes, intentions, and vaccination rates compared to parents in the attention control arm. To prepare for a future full-scale randomized control trial to determine efficacy, our aims were to:
Aim 1: Assess feasibility of the HPVVaxFacts intervention and overall study protocol.
Aim 2: Assess acceptability of the HPVVaxFacts intervention within two urban pediatric clinics.
Aim 3: Explore preliminary efficacy of the HPVVaxFacts intervention on the parent's decision to get their adolescents the HPV vaccination."

METHODS

3a) Description of trial design (such as parallel, factorial) including allocation ratio

Does your paper address CONSORT subitem 3a? *

Copy and paste relevant sections from the manuscript (include quotes in quotation marks "like this" to indicate direct quotes from your manuscript), or elaborate on this item by providing additional information not in the ms, or briefly explain why the item is not applicable/relevant for your study

"A multi-method approach was used to evaluate the feasibility, acceptability, and preliminary efficacy of a mobile web app HPVVaxFacts among parents representing the continuum of hesitancy for vaccination from full acceptance to outright refusal [32]. A pilot, two-arm, multisite randomized control trial (RCT) was conducted at two urban pediatric clinics in Tennessee to determine feasibility and acceptability. Parents in both arms who agreed to follow-up were interviewed to explore acceptability and feasibility of the protocol using a qualitative, descriptive study design. Intervention acceptability and parent-child discussion were examined among parents in the intervention arm only."

"Our study goal was to enroll 70 parents, expecting 10%–15% attrition (e.g., no-show for clinic visit), yielding a final sample of 60–63 parents (28–30 per arm)."

3b) Important changes to methods after trial commencement (such as eligibility criteria), with reasons

Does your paper address CONSORT subitem 3b? *

Copy and paste relevant sections from the manuscript (include quotes in quotation marks "like this" to indicate direct quotes from your manuscript), or elaborate on this item by providing additional information not in the ms, or briefly explain why the item is not applicable/relevant for your study

There were no important changes post rial commencement.

3b-i) Bug fixes, Downtimes, Content Changes

Bug fixes, Downtimes, Content Changes: ehealth systems are often dynamic systems. A description of changes to methods therefore also includes important changes made on the intervention or comparator during the trial (e.g., major bug fixes or changes in the functionality or content) (5-iii) and other “unexpected events” that may have influenced study design such as staff changes, system failures/downtimes, etc. [2].

	1	2	3	4	5	
subitem not at all important	○	○	○	○	◉	essential

Clear selection

Does your paper address subitem 3b-i?

Copy and paste relevant sections from the manuscript (include quotes in quotation marks "like this" to indicate direct quotes from your manuscript), or elaborate on this item by providing additional information not in the ms, or briefly explain why the item is not applicable/relevant for your study

"During the recruitment period, 14 participants were assigned to the nutrition education website due to a mobile web app update performed over 1 week to enhance the feasibility of HPVVaxFacts. Particularly, this update addressed an unresponsive application and added a flashing button to aid parents in submitting survey responses. These individuals were not included in this analysis."

4a) Eligibility criteria for participants

Does your paper address CONSORT subitem 4a? *

Copy and paste relevant sections from the manuscript (include quotes in quotation marks "like this" to indicate direct quotes from your manuscript), or elaborate on this item by providing additional information not in the ms, or briefly explain why the item is not applicable/relevant for your study

"Eligible parents had a child aged 9 to 17 years who were unvaccinated against HPV, had a mobile phone, had an upcoming clinic visit, and spoke English."

4a-i) Computer / Internet literacy

Computer / Internet literacy is often an implicit “de facto” eligibility criterion - this should be explicitly clarified.

	1	2	3	4	5	
subitem not at all important	○	○	○	◉	○	essential

Clear selection

Does your paper address subitem 4a-i?

Copy and paste relevant sections from the manuscript (include quotes in quotation marks "like this" to indicate direct quotes from your manuscript), or elaborate on this item by providing additional information not in the ms, or briefly explain why the item is not applicable/relevant for your study

"This study was conducted with parents who were caregivers for children in two urban pediatric clinics in Tennessee who were representative of the continuum of vaccine hesitancy. Eligible parents had a child aged 9 to 17 years who were unvaccinated against HPV, had a mobile phone, had an upcoming clinic visit, and spoke English."

"Second, some parents may not have participated due to mobile app literacy issues or not having a mobile app, which should be explored in future studies. However, we have begun to take steps to improve inclusion through the provision of HPVVaxFacts in Spanish."

4a-ii) Open vs. closed, web-based vs. face-to-face assessments:

Open vs. closed, web-based vs. face-to-face assessments: Mention how participants were recruited (online vs. offline), e.g., from an open access website or from a clinic, and clarify if this was a purely web-based trial, or there were face-to-face components (as part of the intervention or for assessment), i.e., to what degree got the study team to know the participant. In online-only trials, clarify if participants were quasi-anonymous and whether having multiple identities was possible or whether technical or logistical measures (e.g., cookies, email confirmation, phone calls) were used to detect/prevent these.

	1	2	3	4	5	
subitem not at all important	○	○	○	○	◉	essential

Clear selection

Does your paper address subitem 4a-ii? *

Copy and paste relevant sections from the manuscript (include quotes in quotation marks "like this" to indicate direct quotes from your manuscript), or elaborate on this item by providing additional information not in the ms, or briefly explain why the item is not applicable/relevant for your study

"The clinic sites had different recruitment approaches. Clinic site 1 recruitment was conducted by two trained clinic staff. Clinic site 2 recruitment was done by 2 members of the research team."

"At clinic site one, the parents were first introduced to the study via a post to the clinic’s Facebook page to make parents aware that the study was occurring in their clinic. Eligibility was determined via EHR data by two trained clinic staff at clinic site one, and two trained research staff at clinic site two. The research team was trained by clinic staff to use EHR and eClinicalWorks. If eligible, an IRB-approved text message explaining the study with the invite specifically mentioning HPV vaccination and asking for their participation was sent to parents. If parents text “yes”, a screener was text to parents to confirm eligibility. This was done by trained clinic staff via Phreesia at Clinic Site 1 and research team members via eClinicalWorks (version 12.0.2) at Clinic Site 2. Phreesia is a HIPAA-compliant patient intake management platform that automates the check-in process as well as supports patient-provider communication [41]. Parents received up to three messages on their mobile phone requesting participation."

4a-iii) Information giving during recruitment

Information given during recruitment. Specify how participants were briefed for recruitment and in the informed consent procedures (e.g., publish the informed consent documentation as appendix, see also item X26), as this information may have an effect on user self-selection, user expectation and may also bias results.

	1	2	3	4	5	
subitem not at all important	○	○	○	○	◉	essential

Clear selection

Does your paper address subitem 4a-iii?

Copy and paste relevant sections from the manuscript (include quotes in quotation marks "like this" to indicate direct quotes from your manuscript), or elaborate on this item by providing additional information not in the ms, or briefly explain why the item is not applicable/relevant for your study

"If eligible, an IRB-approved text message explaining the study with the invite specifically mentioning HPV vaccination and asking for their participation was sent to parents. If parents text "yes", a screener was text to parents to confirm eligibility."

"After confirming participant eligibility, parents were sent a REDCap link to their mobile phone to provide e-consent up to 48 hours prior to clinic visit. "

4b) Settings and locations where the data were collected

Does your paper address CONSORT subitem 4b? *

Copy and paste relevant sections from the manuscript (include quotes in quotation marks "like this" to indicate direct quotes from your manuscript), or elaborate on this item by providing additional information not in the ms, or briefly explain why the item is not applicable/relevant for your study

"A pilot, two-arm, multisite randomized control trial (RCT) was conducted at two urban pediatric clinics in Tennessee to determine feasibility and acceptability."

4b-i) Report if outcomes were (self-)assessed through online questionnaires

Clearly report if outcomes were (self-)assessed through online questionnaires (as common in web-based trials) or otherwise.

	1	2	3	4	5	
subitem not at all important	○	○	○	○	◉	essential

Clear selection

Does your paper address subitem 4b-i? *

Copy and paste relevant sections from the manuscript (include quotes in quotation marks "like this" to indicate direct quotes from your manuscript), or elaborate on this item by providing additional information not in the ms, or briefly explain why the item is not applicable/relevant for your study

"The pre-post survey asked parents about their knowledge, attitudes, and intentions for their child to initiate the HPV vaccination process. Socio-demographic data were collected on the pre-survey only. Post study, intervention acceptability and intervention use for parent-child discussion were assessed on the survey of parents in the intervention “HPVVaxFacts” study arm only, and the clinic provided the vaccination status of each child at 12 months post-intervention using the electronic health record."

4b-ii) Report how institutional affiliations are displayed

Report how institutional affiliations are displayed to potential participants [on ehealth media], as affiliations with prestigious hospitals or universities may affect volunteer rates, use, and reactions with regards to an intervention.(Not a required item – describe only if this may bias results)

	1	2	3	4	5	
subitem not at all important	◉	○	○	○	○	essential

Clear selection

Does your paper address subitem 4b-ii?

Copy and paste relevant sections from the manuscript (include quotes in quotation marks "like this" to indicate direct quotes from your manuscript), or elaborate on this item by providing additional information not in the ms, or briefly explain why the item is not applicable/relevant for your study

This does not apply. Because on pediatric group was on site of the institution and the other was identified as a partner to the other site, participants were aware of the institutional affiliation. However, the emphasis was placed on the study purpose and requirements.

5) The interventions for each group with sufficient details to allow replication, including how and when they were actually administered

5-i) Mention names, credential, affiliations of the developers, sponsors, and owners

Mention names, credential, affiliations of the developers, sponsors, and owners [6] (if authors/evaluators are owners or developer of the software, this needs to be declared in a “Conflict of interest” section or mentioned elsewhere in the manuscript).

	1	2	3	4	5	
subitem not at all important	○	○	○	○	◉	essential

Clear selection

Does your paper address subitem 5-i?

Copy and paste relevant sections from the manuscript (include quotes in quotation marks "like this" to indicate direct quotes from your manuscript), or elaborate on this item by providing additional information not in the ms, or briefly explain why the item is not applicable/relevant for your study

Under Conflicts of Interest, "Author JCE is the owner of the web app HPVVaxFacts."

Under acknowledgements "The authors would also like to thank 233 analytics who developed and maintained the intervention."

5-ii) Describe the history/development process

Describe the history/development process of the application and previous formative evaluations (e.g., focus groups, usability testing), as these will have an impact on adoption/use rates and help with interpreting results.

	1	2	3	4	5	
subitem not at all important	○	○	◉	○	○	essential

Clear selection

Does your paper address subitem 5-ii?

Copy and paste relevant sections from the manuscript (include quotes in quotation marks "like this" to indicate direct quotes from your manuscript), or elaborate on this item by providing additional information not in the ms, or briefly explain why the item is not applicable/relevant for your study

This is not the primary objective for this manuscript. However, we link reviewers to the manuscript describing the formative research process for the intervention's development. "See Cunningham-Erves et al. 36 for full description of HPVVaxFacts development."

5-iii) Revisions and updating

Revisions and updating. Clearly mention the date and/or version number of the application/intervention (and comparator, if applicable) evaluated, or describe whether the intervention underwent major changes during the evaluation process, or whether the development and/or content was "frozen" during the trial. Describe dynamic components such as news feeds or changing content which may have an impact on the replicability of the intervention (for unexpected events see item 3b).

	1	2	3	4	5	
subitem not at all important	○	○	○	○	◉	essential

Clear selection

Does your paper address subitem 5-iii?

Copy and paste relevant sections from the manuscript (include quotes in quotation marks "like this" to indicate direct quotes from your manuscript), or elaborate on this item by providing additional information not in the ms, or briefly explain why the item is not applicable/relevant for your study

"During the recruitment period, 14 participants were assigned to the nutrition education website due to a mobile web app update performed over 1 week to enhance the feasibility of HPVVaxFacts. Particularly, this update addressed an unresponsive application and added a flashing button to aid parents in submitting survey responses. These individuals were not included in this analysis."

5-iv) Quality assurance methods

Provide information on quality assurance methods to ensure accuracy and quality of information provided [1], if applicable.

	1	2	3	4	5	
subitem not at all important	○	○	○	○	◉	essential

Clear selection

Does your paper address subitem 5-iv?

Copy and paste relevant sections from the manuscript (include quotes in quotation marks "like this" to indicate direct quotes from your manuscript), or elaborate on this item by providing additional information not in the ms, or briefly explain why the item is not applicable/relevant for your study

This information is described in the manuscript on formative research development of the tool. We offered this manuscript for further detail. "See Cunningham-Erves et al. 36 for full description of HPVVaxFacts development."

5-v) Ensure replicability by publishing the source code, and/or providing screenshots/screen-capture video, and/or providing flowcharts of the algorithms used

Ensure replicability by publishing the source code, and/or providing screenshots/screen-capture video, and/or providing flowcharts of the algorithms used. Replicability (i.e., other researchers should in principle be able to replicate the study) is a hallmark of scientific reporting.

	1	2	3	4	5	
subitem not at all important	◉	○	○	○	○	essential

Clear selection

Does your paper address subitem 5-v?

Copy and paste relevant sections from the manuscript (include quotes in quotation marks "like this" to indicate direct quotes from your manuscript), or elaborate on this item by providing additional information not in the ms, or briefly explain why the item is not applicable/relevant for your study

Because the researchers plan to continue testing of this tool for efficacy, we do not provide the source code and/or algorithms. However, screenshots of the tool can be found in the formative research study in which the reference is cited in text.

5-vi) Digital preservation

Digital preservation: Provide the URL of the application, but as the intervention is likely to change or disappear over the course of the years; also make sure the intervention is archived (Internet Archive, webcitation.org, and/or publishing the source code or screenshots/videos alongside the article). As pages behind login screens cannot be archived, consider creating demo pages which are accessible without login.

	1	2	3	4	5	
subitem not at all important	(•)	( )	( )	( )	( )	essential

Clear selection

Does your paper address subitem 5-vi?

Copy and paste relevant sections from the manuscript (include quotes in quotation marks "like this" to indicate direct quotes from your manuscript), or elaborate on this item by providing additional information not in the ms, or briefly explain why the item is not applicable/relevant for your study

The authors will create demo pages once this tool has been tested for efficacy in future studies.

5-vii) Access

Access: Describe how participants accessed the application, in what setting/context, if they had to pay (or were paid) or not, whether they had to be a member of specific group. If known, describe how participants obtained "access to the platform and Internet" [1]. To ensure access for editors/reviewers/readers, consider to provide a "backdoor" login account or demo mode for reviewers/readers to explore the application (also important for archiving purposes, see vi).

	1	2	3	4	5	
subitem not at all important	( )	( )	( )	( )	(•)	essential

Clear selection

Does your paper address subitem 5-vii? *

Copy and paste relevant sections from the manuscript (include quotes in quotation marks "like this" to indicate direct quotes from your manuscript), or elaborate on this item by providing additional information not in the ms, or briefly explain why the item is not applicable/relevant for your study

"If randomized to the intervention arm, parents were sent a personalized link to the pre-survey to assess knowledge and intention, and then went to the mobile web app HPVVaxFacts to assess and rank their top three attitudes (i.e., concerns)."

Note: App is currently being updated and not available.

5-viii) Mode of delivery, features/functionalities/components of the intervention and comparator, and the theoretical framework

Describe mode of delivery, features/functionalities/components of the intervention and comparator, and the theoretical framework [6] used to design them (instructional strategy [1], behaviour change techniques, persuasive features, etc., see e.g., [7, 8] for terminology). This includes an in-depth description of the content (including where it is coming from and who developed it) [1]," whether [and how] it is tailored to individual circumstances and allows users to track their progress and receive feedback" [6]. This also includes a description of communication delivery channels and – if computer-mediated communication is a component – whether communication was synchronous or asynchronous [6]. It also includes information on presentation strategies [1], including page design principles, average amount of text on pages, presence of hyperlinks to other resources, etc. [1].

	1	2	3	4	5	
subitem not at all important	○	○	○	○	◉	essential

Clear selection

Does your paper address subitem 5-viii? *

Copy and paste relevant sections from the manuscript (include quotes in quotation marks "like this" to indicate direct quotes from your manuscript), or elaborate on this item by providing additional information not in the ms, or briefly explain why the item is not applicable/relevant for your study

"HPVVaxFacts provides individually tailored education on HPV and the vaccine to parents [36]. Education and video content of each concern was developed using the Health Belief Model and Theory of Reasoned Action (Figure 1). To tailor the education in the app, each parent answered a 10-item baseline survey adapted from the Vaccination Confidence Scale [37] to identify parental concerns and the survey responses are then ranked to identify the top three parental concerns. Parents can change their ranking if they disagree. Parents then review the content (i.e., educational information, provider's video, questions for the provider, and links to other credible sources verifying content) associated with each concern. An adolescent corner is available for parents to share with and/or have an optional discussion with their child about each concern. Last, parents could submit concerns or additional needs via the provision of feedback. See Cunningham-Erves et al. [36] for full description of HPVVaxFacts development."

We also provide a reference to the formative research process for development to gain more insight on the content and delivery processes.

5-ix) Describe use parameters

Describe use parameters (e.g., intended "doses" and optimal timing for use). Clarify what instructions or recommendations were given to the user, e.g., regarding timing, frequency, heaviness of use, if any, or was the intervention used ad libitum.

	1	2	3	4	5	
subitem not at all important	○	○	○	◉	○	essential

Clear selection

Does your paper address subitem 5-ix?

Copy and paste relevant sections from the manuscript (include quotes in quotation marks "like this" to indicate direct quotes from your manuscript), or elaborate on this item by providing additional information not in the ms, or briefly explain why the item is not applicable/relevant for your study

"Parents were asked to view HPV or nutrition programs at least once prior to their upcoming clinic visit."

5-x) Clarify the level of human involvement

Clarify the level of human involvement (care providers or health professionals, also technical assistance) in the e-intervention or as co-intervention (detail number and expertise of professionals involved, if any, as well as "type of assistance offered, the timing and frequency of the support, how it is initiated, and the medium by which the assistance is delivered". It may be necessary to distinguish between the level of human involvement required for the trial, and the level of human involvement required for a routine application outside of a RCT setting (discuss under item 21 – generalizability).

	1	2	3	4	5	
subitem not at all important	○	○	○	○	●	essential

Clear selection

Does your paper address subitem 5-x?

Copy and paste relevant sections from the manuscript (include quotes in quotation marks "like this" to indicate direct quotes from your manuscript), or elaborate on this item by providing additional information not in the ms, or briefly explain why the item is not applicable/relevant for your study

We describe how the degree of clinical staff involvement in study recruitment in our study, and their role in the process.

5-xi) Report any prompts/reminders used

Report any prompts/reminders used: Clarify if there were prompts (letters, emails, phone calls, SMS) to use the application, what triggered them, frequency etc. It may be necessary to distinguish between the level of prompts/reminders required for the trial, and the level of prompts/reminders for a routine application outside of a RCT setting (discuss under item 21 – generalizability).

	1	2	3	4	5	
subitem not at all important	○	○	○	○	◉	essential

Clear selection

Does your paper address subitem 5-xi? *

Copy and paste relevant sections from the manuscript (include quotes in quotation marks "like this" to indicate direct quotes from your manuscript), or elaborate on this item by providing additional information not in the ms, or briefly explain why the item is not applicable/relevant for your study

"If any participant did not complete a survey or view the mobile web app, up to 3 reminders were sent for completion."

5-xii) Describe any co-interventions (incl. training/support)

Describe any co-interventions (incl. training/support): Clearly state any interventions that are provided in addition to the targeted eHealth intervention, as ehealth intervention may not be designed as stand-alone intervention. This includes training sessions and support [1]. It may be necessary to distinguish between the level of training required for the trial, and the level of training for a routine application outside of a RCT setting (discuss under item 21 – generalizability.

	1	2	3	4	5	
subitem not at all important	◉	○	○	○	○	essential

Clear selection

Does your paper address subitem 5-xii? *

Copy and paste relevant sections from the manuscript (include quotes in quotation marks "like this" to indicate direct quotes from your manuscript), or elaborate on this item by providing additional information not in the ms, or briefly explain why the item is not applicable/relevant for your study

We did not have a co-intervention so this is not applicable.

6a) Completely defined pre-specified primary and secondary outcome measures, including how and when they were assessed

Does your paper address CONSORT subitem 6a? *

Copy and paste relevant sections from the manuscript (include quotes in quotation marks "like this" to indicate direct quotes from your manuscript), or elaborate on this item by providing additional information not in the ms, or briefly explain why the item is not applicable/relevant for your study

"Outcomes. The clinic provided the vaccination status of each child at 12 months post-intervention using the electronic health record (Primary Outcome). The pre-post survey asked parents about their knowledge, attitudes, and intentions for their child to initiate the HPV vaccination process. Intervention acceptability and intervention use for parent-child discussion were assessed on the post-survey of parents in the intervention, “HPVVaxFacts” study arm only (Secondary Outcomes). Socio-demographic data were collected on the pre-survey only (Covariates)."

6a-i) Online questionnaires: describe if they were validated for online use and apply CHERRIES items to describe how the questionnaires were designed/deployed

If outcomes were obtained through online questionnaires, describe if they were validated for online use and apply CHERRIES items to describe how the questionnaires were designed/deployed [9].

	1	2	3	4	5	
subitem not at all important	○	○	○	◉	○	essential

Clear selection

Does your paper address subitem 6a-i?

Copy and paste relevant sections from manuscript text

The scales were validated for online use in prior studies. Each scale in text is described with an associated reference.

6a-ii) Describe whether and how “use” (including intensity of use/dosage) was defined/measured/monitored

Describe whether and how “use” (including intensity of use/dosage) was defined/measured/monitored (logins, logfile analysis, etc.). Use/adoption metrics are important process outcomes that should be reported in any ehealth trial.

	1	2	3	4	5	
subitem not at all important	○	○	○	○	◉	essential

Clear selection

Does your paper address subitem 6a-ii?

Copy and paste relevant sections from manuscript text

"Thirty-six parents were randomly assigned to and logged into the HPVVaxFacts, of which seven did not view the mobile web app. Of those who viewed the mobile web app (81%), two did not complete the post-survey. These participants were excluded with a retention rate of 75%. Thirty-five people were randomly assigned to the attention control group. We were unable to determine if participants viewed the nutrition education website. However, two did not complete the post-survey with a retention rate of 94% (Figure 3)."

6a-iii) Describe whether, how, and when qualitative feedback from participants was obtained

Describe whether, how, and when qualitative feedback from participants was obtained (e.g., through emails, feedback forms, interviews, focus groups).

	1	2	3	4	5	
subitem not at all important	○	○	○	○	◉	essential

Clear selection

Does your paper address subitem 6a-iii?

Copy and paste relevant sections from manuscript text

"Interviews were conducted up to 9 months post-intervention based on parent availability. On the post survey, 15 in the intervention study arm and 18 in nutrition attention control agreed to be contacted for follow-up interviews. Of these participants, nine in the intervention study arm and 10 in the nutrition attention control completed an interview. Other parents either could not be reached (n=9) to complete or later decided not to complete the interview (n=5)."

6b) Any changes to trial outcomes after the trial commenced, with reasons

Does your paper address CONSORT subitem 6b? *

Copy and paste relevant sections from the manuscript (include quotes in quotation marks "like this" to indicate direct quotes from your manuscript), or elaborate on this item by providing additional information not in the ms, or briefly explain why the item is not applicable/relevant for your study

There were no changes in trial outcomes after the trial commenced.

7a) How sample size was determined

NPT: When applicable, details of whether and how the clustering by care provides or centers was addressed

7a-i) Describe whether and how expected attrition was taken into account when calculating the sample size

Describe whether and how expected attrition was taken into account when calculating the sample size.

	1	2	3	4	5	
subitem not at all important	○	○	○	○	◉	essential

Clear selection

Does your paper address subitem 7a-i?

Copy and paste relevant sections from manuscript title (include quotes in quotation marks "like this" to indicate direct quotes from your manuscript), or elaborate on this item by providing additional information not in the ms, or briefly explain why the item is not applicable/relevant for your study

"GPower 3.1 program was used to conduct power analysis to determine the sample size. The necessary sample size for the pilot trial was calculated using a one-sided 80% confidence interval (CI) and the expected effect size of the future randomized control trial [42], based on the expected proportion in the attention control arm (0.20–0.30) and the clinically meaningful difference to be observed in the intervention arm in the future RCT (at least 0.10 higher) [42,43]. A total pilot sample size of 46–60 after attrition was deemed sufficient. Our study goal was to enroll 70 parents, expecting 10%–15% attrition (e.g., no-show for clinic visit), yielding a final sample of 60–63 parents (28–30 per arm)."

7b) When applicable, explanation of any interim analyses and stopping guidelines

Does your paper address CONSORT subitem 7b? *

Copy and paste relevant sections from the manuscript (include quotes in quotation marks "like this" to indicate direct quotes from your manuscript), or elaborate on this item by providing additional information not in the ms, or briefly explain why the item is not applicable/relevant for your study

This is not applicable to this study.

8a) Method used to generate the random allocation sequence

NPT: When applicable, how care providers were allocated to each trial group

Does your paper address CONSORT subitem 8a? *

Copy and paste relevant sections from the manuscript (include quotes in quotation marks "like this" to indicate direct quotes from your manuscript), or elaborate on this item by providing additional information not in the ms, or briefly explain why the item is not applicable/relevant for your study

"The biostatistician (co-author DL) generated two randomization lists prior to study implementation. Using blocked randomization, randomization was conducted with blocks of 10 (using n=10 from each group). Group labels (denoted as 1 or 2) were generated in Stata version 17."

8b) Type of randomisation; details of any restriction (such as blocking and block size)

Does your paper address CONSORT subitem 8b? *

Copy and paste relevant sections from the manuscript (include quotes in quotation marks "like this" to indicate direct quotes from your manuscript), or elaborate on this item by providing additional information not in the ms, or briefly explain why the item is not applicable/relevant for your study

"Using blocked randomization, randomization was conducted with blocks of 10 (using n=10 from each group). Group labels (denoted as 1 or 2) were generated in Stata version 17."

9) Mechanism used to implement the random allocation sequence (such as sequentially numbered containers), describing any steps taken to conceal the sequence until interventions were assigned

Does your paper address CONSORT subitem 9? *

Copy and paste relevant sections from the manuscript (include quotes in quotation marks "like this" to indicate direct quotes from your manuscript), or elaborate on this item by providing additional information not in the ms, or briefly explain why the item is not applicable/relevant for your study

"Using blocked randomization, randomization was conducted with blocks of 10 (using n=10 from each group). Group labels (denoted as 1 or 2) were generated in Stata version 17. "

10) Who generated the random allocation sequence, who enrolled participants, and who assigned participants to interventions

Does your paper address CONSORT subitem 10? *

Copy and paste relevant sections from the manuscript (include quotes in quotation marks "like this" to indicate direct quotes from your manuscript), or elaborate on this item by providing additional information not in the ms, or briefly explain why the item is not applicable/relevant for your study

"The biostatistician (co-author DL) generated two randomization lists prior to study implementation."

11a) If done, who was blinded after assignment to interventions (for example, participants, care providers, those assessing outcomes) and how

NPT: Whether or not administering co-interventions were blinded to group assignment

11a-i) Specify who was blinded, and who wasn't

Specify who was blinded, and who wasn't. Usually, in web-based trials it is not possible to blind the participants [1, 3] (this should be clearly acknowledged), but it may be possible to blind outcome assessors, those doing data analysis or those administering co-interventions (if any).

	1	2	3	4	5	
subitem not at all important	○	○	○	○	◉	essential

Clear selection

Does your paper address subitem 11a-i? *

Copy and paste relevant sections from the manuscript (include quotes in quotation marks "like this" to indicate direct quotes from your manuscript), or elaborate on this item by providing additional information not in the ms, or briefly explain why the item is not applicable/relevant for your study

"While parents were not blinded to the study arm assignment, providers were blinded."

11a-ii) Discuss e.g., whether participants knew which intervention was the "intervention of interest" and which one was the "comparator"

Informed consent procedures (4a-ii) can create biases and certain expectations - discuss e.g., whether participants knew which intervention was the "intervention of interest" and which one was the "comparator".

	1	2	3	4	5	
subitem not at all important	○	○	○	○	◉	essential

Clear selection

Does your paper address subitem 11a-ii?

Copy and paste relevant sections from the manuscript (include quotes in quotation marks "like this" to indicate direct quotes from your manuscript), or elaborate on this item by providing additional information not in the ms, or briefly explain why the item is not applicable/relevant for your study

"While parents were functionally not unblinded, they could deduce their study arm assignment. Providers were blinded."

"After confirming participant eligibility, parents were sent a REDCap link to their mobile phone to provide e-consent up to 48 hours prior to clinic visit. They were assigned to either the intervention or attention control group post-consent based on the computer-generated randomization numbers list by a trained, research team member with a social science background (Lead Author: JCE)."

"Last, it is possible that the attention control of nutrition education could have had an effect on parents to accept and/or obtain the initial dose of the HPV vaccination for their child."

11b) If relevant, description of the similarity of interventions

(this item is usually not relevant for ehealth trials as it refers to similarity of a placebo or sham intervention to a active medication/intervention)

Does your paper address CONSORT subitem 11b? *

Copy and paste relevant sections from the manuscript (include quotes in quotation marks "like this" to indicate direct quotes from your manuscript), or elaborate on this item by providing additional information not in the ms, or briefly explain why the item is not applicable/relevant for your study

We have an ehealth trial and this is not applicable.

12a) Statistical methods used to compare groups for primary and secondary outcomes

NPT: When applicable, details of whether and how the clustering by care providers or centers was addressed

Does your paper address CONSORT subitem 12a? *

Copy and paste relevant sections from the manuscript (include quotes in quotation marks "like this" to indicate direct quotes from your manuscript), or elaborate on this item by providing additional information not in the ms, or briefly explain why the item is not applicable/relevant for your study

"Comparisons between randomized study arms were conducted using statistical tests as appropriate, including Fisher's exact test of chi-squared tests for categorical variables and Wilcoxon rank sum tests or t-tests for continuous variables. Because participants were excluded after randomization due to incomplete intervention exposure or missing outcome data, exploratory statistical comparisons between study arms were performed to evaluate potential differences in baseline characteristics. Comparison of pre-post scores of HPV vaccine knowledge, attitudes, and intention by study arm was performed using exploratory visual analysis."

12a-i) Imputation techniques to deal with attrition / missing values

Imputation techniques to deal with attrition / missing values: Not all participants will use the intervention/comparator as intended and attrition is typically high in ehealth trials. Specify how participants who did not use the application or dropped out from the trial were treated in the statistical analysis (a complete case analysis is strongly discouraged, and simple imputation techniques such as LOCF may also be problematic [4]).

	1	2	3	4	5	
subitem not at all important	○	○	○	○	◉	essential

Clear selection

Does your paper address subitem 12a-i? *

Copy and paste relevant sections from the manuscript (include quotes in quotation marks "like this" to indicate direct quotes from your manuscript), or elaborate on this item by providing additional information not in the ms, or briefly explain why the item is not applicable/relevant for your study

"Because participants were excluded after randomization due to incomplete intervention exposure or missing outcome data, exploratory statistical comparisons between study arms were performed to evaluate potential differences in baseline characteristics."

"The data needed for study evaluation were available with less than 5% missing data from the survey, EHR for child's age and vaccine status, and HPVVaxFacts data log tracking of parent concerns and ranking top three HPV vaccine concerns."

12b) Methods for additional analyses, such as subgroup analyses and adjusted analyses

Does your paper address CONSORT subitem 12b? *

Copy and paste relevant sections from the manuscript (include quotes in quotation marks "like this" to indicate direct quotes from your manuscript), or elaborate on this item by providing additional information not in the ms, or briefly explain why the item is not applicable/relevant for your study

This was a small pilot RCT, so we did not do subgroup analyses.

X26) REB/IRB Approval and Ethical Considerations [recommended as subheading under "Methods"] (not a CONSORT item)

X26-i) Comment on ethics committee approval

	1	2	3	4	5	
subitem not at all important	○	○	○	○	◉	essential

Clear selection

Does your paper address subitem X26-i?

Copy and paste relevant sections from the manuscript (include quotes in quotation marks "like this" to indicate direct quotes from your manuscript), or elaborate on this item by providing additional information not in the ms, or briefly explain why the item is not applicable/relevant for your study

"The Institutional Review Board of Meharry Medical College (Protocol Number: 18-12-890) approved this study as expedited as it was considered no more than minimal risk. The study was registered at ClinicalTrials.gov (NCT# NCT04380623) and performed in line with the principles of the Declaration of Helsinki. Electronic consent was obtained from participants using REDCap [34,35] for the pilot RCT, and verbal consent for the semi-structured interviews. To protect participant information, survey and interview data were stored via REDCap and on a password protected server. Data were de-identified prior to provision to statisticians and research team members for data analysis. Dissemination of results was aggregate and anonymous. Participants were compensated with a $30 gift card for pilot study participation, and a $20 gift card for completing a follow-up interview."

x26-ii) Outline informed consent procedures

Outline informed consent procedures e.g., if consent was obtained offline or online (how? Checkbox, etc.?), and what information was provided (see 4a-ii). See [6] for some items to be included in informed consent documents.

	1	2	3	4	5	
subitem not at all important	○	○	○	○	◉	essential

Clear selection

Does your paper address subitem X26-ii?

Copy and paste relevant sections from the manuscript (include quotes in quotation marks "like this" to indicate direct quotes from your manuscript), or elaborate on this item by providing additional information not in the ms, or briefly explain why the item is not applicable/relevant for your study

"After confirming participant eligibility, parents were sent a REDCap link to their mobile phone to provide e-consent up to 48 hours prior to clinic visit."

X26-iii) Safety and security procedures

Safety and security procedures, incl. privacy considerations, and any steps taken to reduce the likelihood or detection of harm (e.g., education and training, availability of a hotline)

	1	2	3	4	5	
subitem not at all important	○	○	○	○	◉	essential

Clear selection

Does your paper address subitem X26-iii?

Copy and paste relevant sections from the manuscript (include quotes in quotation marks "like this" to indicate direct quotes from your manuscript), or elaborate on this item by providing additional information not in the ms, or briefly explain why the item is not applicable/relevant for your study

"Participants were allowed to provide feedback on the HPVVaxFacts mobile web app to determine if important concerns were missing from the app, which suggests missed questions on the attitudes (i.e., concern) scale."

"Last, parents could submit concerns or additional needs via the provision of feedback."

RESULTS

13a) For each group, the numbers of participants who were randomly assigned, received intended treatment, and were analysed for the primary outcome
NPT: The number of care providers or centers performing the intervention in each group and the number of patients treated by each care provider in each center

Does your paper address CONSORT subitem 13a? *

Copy and paste relevant sections from the manuscript (include quotes in quotation marks "like this" to indicate direct quotes from your manuscript), or elaborate on this item by providing additional information not in the ms, or briefly explain why the item is not applicable/relevant for your study

"Of the 194 assessed for eligibility, 63 were ineligible. Twenty-one (16%) declined to participate with an enrollment rate of 84%. Nine more later refused, increasing the refusal rate to 23% with an enrollment rate of 40.3%. We recruited 110 participants who consented and enrolled in the study (107 were recruited from clinic site one and three from clinic site two). Parents enrolled 15 to 20 minutes up to 24 hours prior to clinic visit. Thirty-six parents were randomly assigned to and logged into the HPVVaxFacts, of which seven did not view the mobile web app. Of those who viewed the mobile web app (81%), two did not complete the post-survey. These participants were excluded with a retention rate of 75%. Thirty-five people were randomly assigned to the attention control group."

13b) For each group, losses and exclusions after randomisation, together with reasons

Does your paper address CONSORT subitem 13b? (NOTE: Preferably, this is shown in a CONSORT flow diagram) *

Copy and paste relevant sections from the manuscript (include quotes in quotation marks "like this" to indicate direct quotes from your manuscript), or elaborate on this item by providing additional information not in the ms, or briefly explain why the item is not applicable/relevant for your study

"Parents enrolled 15 to 20 minutes up to 24 hours prior to clinic visit. Thirty-six parents were randomly assigned to and logged into the HPVVaxFacts, of which seven did not view the mobile web app. Of those who viewed the mobile web app (81%), two did not complete the post-survey. These participants were excluded with a retention rate of 75%. Thirty-five people were randomly assigned to the attention control group."

13b-i) Attrition diagram

Strongly recommended: An attrition diagram (e.g., proportion of participants still logging in or using the intervention/comparator in each group plotted over time, similar to a survival curve) or other figures or tables demonstrating usage/dose/engagement.

	1	2	3	4	5	
subitem not at all important	○	○	◉	○	○	essential

Clear selection

Does your paper address subitem 13b-i?

Copy and paste relevant sections from the manuscript or cite the figure number if applicable (include quotes in quotation marks "like this" to indicate direct quotes from your manuscript), or elaborate on this item by providing additional information not in the ms, or briefly explain why the item is not applicable/relevant for your study

"Parents enrolled 15 to 20 minutes up to 24 hours prior to clinic visit. Thirty-six parents were randomly assigned to and logged into the HPVVaxFacts, of which seven did not view the mobile web app. Of those who viewed the mobile web app (81%), two did not complete the post-survey. These participants were excluded with a retention rate of 75%. Thirty-five people were randomly assigned to the attention control group."

14a) Dates defining the periods of recruitment and follow-up

Does your paper address CONSORT subitem 14a? *

Copy and paste relevant sections from the manuscript (include quotes in quotation marks "like this" to indicate direct quotes from your manuscript), or elaborate on this item by providing additional information not in the ms, or briefly explain why the item is not applicable/relevant for your study

"At clinic site one, quantitative data were collected between June and September 2023. At clinic site two, data collection occurred one week in August 2023 for the back-to-school immunization week. The data collection time frame was recommended by clinic staff because this time had highest volume of unvaccinated patients despite their high HPV vaccination rates in-clinic."

"Interviews were conducted up to 9 months post-intervention based on parent availability. "

14a-i) Indicate if critical “secular events” fell into the study period

Indicate if critical “secular events” fell into the study period, e.g., significant changes in Internet resources available or “changes in computer hardware or Internet delivery resources”

	1	2	3	4	5	
subitem not at all important	(●)	○	○	○	○	essential

Clear selection

Does your paper address subitem 14a-i?

Copy and paste relevant sections from the manuscript (include quotes in quotation marks "like this" to indicate direct quotes from your manuscript), or elaborate on this item by providing additional information not in the ms, or briefly explain why the item is not applicable/relevant for your study

Secular events did not occur as far as we know. So we did not add any information.

14b) Why the trial ended or was stopped (early)

Does your paper address CONSORT subitem 14b? *

Copy and paste relevant sections from the manuscript (include quotes in quotation marks "like this" to indicate direct quotes from your manuscript), or elaborate on this item by providing additional information not in the ms, or briefly explain why the item is not applicable/relevant for your study

"At clinic site one, quantitative data were collected between June and September 2023. At clinic site two, data collection occurred one week in August 2023 for the back-to-school immunization week. The data collection time frame was recommended by clinic staff because this time had the highest volume of unvaccinated patients despite their high HPV vaccination rates in-clinic."

15) A table showing baseline demographic and clinical characteristics for each group

NPT: When applicable, a description of care providers (case volume, qualification, expertise, etc.) and centers (volume) in each group

Does your paper address CONSORT subitem 15? *

Copy and paste relevant sections from the manuscript (include quotes in quotation marks "like this" to indicate direct quotes from your manuscript), or elaborate on this item by providing additional information not in the ms, or briefly explain why the item is not applicable/relevant for your study

We have a table showing baseline demographics for each group.

15-i) Report demographics associated with digital divide issues

In ehealth trials it is particularly important to report demographics associated with digital divide issues, such as age, education, gender, social-economic status, computer/Internet/ehealth literacy of the participants, if known.

	1	2	3	4	5	
subitem not at all important	(●)	○	○	○	○	essential

Clear selection

Does your paper address subitem 15-i? *

Copy and paste relevant sections from the manuscript (include quotes in quotation marks "like this" to indicate direct quotes from your manuscript), or elaborate on this item by providing additional information not in the ms, or briefly explain why the item is not applicable/relevant for your study

We did not collect this data and have cited this as a limitation and should be reviewed in future studies.

"Second, some parents may not have participated due to mobile app literacy issues or not having a mobile app, which should be explored in future studies. However, we have begun to take steps to improve inclusion through the provision of HPVVaxFacts in Spanish."

16) For each group, number of participants (denominator) included in each analysis and whether the analysis was by original assigned groups

16-i) Report multiple “denominators” and provide definitions

Report multiple “denominators” and provide definitions: Report N’s (and effect sizes) “across a range of study participation [and use] thresholds” [1], e.g., N exposed, N consented, N used more than x times, N used more than y weeks, N participants “used” the intervention/comparator at specific pre-defined time points of interest (in absolute and relative numbers per group). Always clearly define “use” of the intervention.

	1	2	3	4	5	
subitem not at all important	◉	○	○	○	○	essential

Clear selection

Does your paper address subitem 16-i? *

Copy and paste relevant sections from the manuscript (include quotes in quotation marks "like this" to indicate direct quotes from your manuscript), or elaborate on this item by providing additional information not in the ms, or briefly explain why the item is not applicable/relevant for your study

This information has been provided in the consort diagram in the manuscript.

16-ii) Primary analysis should be intent-to-treat

Primary analysis should be intent-to-treat, secondary analyses could include comparing only “users”, with the appropriate caveats that this is no longer a randomized sample (see 18-i).

	1	2	3	4	5	
subitem not at all important	◉	○	○	○	○	essential

Clear selection

Does your paper address subitem 16-ii?

Copy and paste relevant sections from the manuscript (include quotes in quotation marks "like this" to indicate direct quotes from your manuscript), or elaborate on this item by providing additional information not in the ms, or briefly explain why the item is not applicable/relevant for your study

item not applicable as the effect of the treatment as actually received was the study purpose.

17a) For each primary and secondary outcome, results for each group, and the estimated effect size and its precision (such as 95% confidence interval)

Does your paper address CONSORT subitem 17a? *

Copy and paste relevant sections from the manuscript (include quotes in quotation marks "like this" to indicate direct quotes from your manuscript), or elaborate on this item by providing additional information not in the ms, or briefly explain why the item is not applicable/relevant for your study

We provide the results for primary and secondary outcomes for the individuals in the control and intervention group including the confidence interval.

17a-i) Presentation of process outcomes such as metrics of use and intensity of use

In addition to primary/secondary (clinical) outcomes, the presentation of process outcomes such as metrics of use and intensity of use (dose, exposure) and their operational definitions is critical. This does not only refer to metrics of attrition (13-b) (often a binary variable), but also to more continuous exposure metrics such as "average session length". These must be accompanied by a technical description how a metric like a "session" is defined (e.g., timeout after idle time) [1] (report under item 6a).

	1	2	3	4	5	
subitem not at all important	○	○	○	●	○	essential

Clear selection

Does your paper address subitem 17a-i?

Copy and paste relevant sections from the manuscript (include quotes in quotation marks "like this" to indicate direct quotes from your manuscript), or elaborate on this item by providing additional information not in the ms, or briefly explain why the item is not applicable/relevant for your study

"We documented if participants complied with assessment and data collection protocols. This included: 1) completion rates of the pre-post survey; 2) mobile web app tracking of parent concerns and ranked top three concerns by date and time; and 3) the ability to obtain the necessary data from clinic administrative records, particularly age and HPV vaccination status."

"Eighth, some mobile web app components (i.e., average session length) were not able to be tracked due to financial constraints. However, it is currently being updated to address this limitation."

17b) For binary outcomes, presentation of both absolute and relative effect sizes is recommended

Does your paper address CONSORT subitem 17b? *

Copy and paste relevant sections from the manuscript (include quotes in quotation marks "like this" to indicate direct quotes from your manuscript), or elaborate on this item by providing additional information not in the ms, or briefly explain why the item is not applicable/relevant for your study

For Binary Outcome, we reported the percentage and p-value of participants who initiated the HPV Vaccination post-study in both groups. Because our study was a small multimethod pilot RCT with limited sample size, absolute (risk difference) and relative effect sizes (odds ratios and risk ratios) were not calculated to avoid overestimation of the intervention effect size.

18) Results of any other analyses performed, including subgroup analyses and adjusted analyses, distinguishing pre-specified from exploratory

Does your paper address CONSORT subitem 18? *

Copy and paste relevant sections from the manuscript (include quotes in quotation marks "like this" to indicate direct quotes from your manuscript), or elaborate on this item by providing additional information not in the ms, or briefly explain why the item is not applicable/relevant for your study

This does not apply to our study.

18-i) Subgroup analysis of comparing only users

A subgroup analysis of comparing only users is not uncommon in ehealth trials, but if done, it must be stressed that this is a self-selected sample and no longer an unbiased sample from a randomized trial (see 16-iii).

	1	2	3	4	5	
subitem not at all important	◉	○	○	○	○	essential

Clear selection

Does your paper address subitem 18-i?

Copy and paste relevant sections from the manuscript (include quotes in quotation marks "like this" to indicate direct quotes from your manuscript), or elaborate on this item by providing additional information not in the ms, or briefly explain why the item is not applicable/relevant for your study

This does not apply to our study.

19) All important harms or unintended effects in each group
(for specific guidance see CONSORT for harms)

Does your paper address CONSORT subitem 19? *

Copy and paste relevant sections from the manuscript (include quotes in quotation marks "like this" to indicate direct quotes from your manuscript), or elaborate on this item by providing additional information not in the ms, or briefly explain why the item is not applicable/relevant for your study

This was not reported in our study so not applicable.

19-i) Include privacy breaches, technical problems

Include privacy breaches, technical problems. This does not only include physical "harm" to participants, but also incidents such as perceived or real privacy breaches [1], technical problems, and other unexpected/unintended incidents. "Unintended effects" also includes unintended positive effects [2].

	1	2	3	4	5	
subitem not at all important	○	○	○	○	◉	essential

Clear selection

Does your paper address subitem 19-i?

Copy and paste relevant sections from the manuscript (include quotes in quotation marks "like this" to indicate direct quotes from your manuscript), or elaborate on this item by providing additional information not in the ms, or briefly explain why the item is not applicable/relevant for your study

This was not applicable to this study.

19-ii) Include qualitative feedback from participants or observations from staff/researchers

Include qualitative feedback from participants or observations from staff/researchers, if available, on strengths and shortcomings of the application, especially if they point to unintended/unexpected effects or uses. This includes (if available) reasons for why people did or did not use the application as intended by the developers.

	1	2	3	4	5	
subitem not at all important	○	○	○	○	●	essential

Clear selection

Does your paper address subitem 19-ii?

Copy and paste relevant sections from the manuscript (include quotes in quotation marks "like this" to indicate direct quotes from your manuscript), or elaborate on this item by providing additional information not in the ms, or briefly explain why the item is not applicable/relevant for your study

"Particularly, as top benefit was offering education preclinic visits to their patients."
"Another perceived benefit was the increased vaccination rates in the clinic as a result of the intervention."
"Regarding barriers, providers and clinic staff highlighted the need to improve logistics for large scale implementation."

DISCUSSION

22) Interpretation consistent with results, balancing benefits and harms, and considering other relevant evidence

NPT: In addition, take into account the choice of the comparator, lack of or partial blinding, and unequal expertise of care providers or centers in each group

22-i) Restate study questions and summarize the answers suggested by the data, starting with primary outcomes and process outcomes (use)

Restate study questions and summarize the answers suggested by the data, starting with primary outcomes and process outcomes (use).

	1	2	3	4	5	
subitem not at all important	○	○	○	○	◉	essential

Clear selection

Does your paper address subitem 22-i? *

Copy and paste relevant sections from the manuscript (include quotes in quotation marks "like this" to indicate direct quotes from your manuscript), or elaborate on this item by providing additional information not in the ms, or briefly explain why the item is not applicable/relevant for your study

"Our study assessed the feasibility, and acceptability, and preliminary efficacy of the theory-informed, mobile web app HPVVaxFacts that seeks to increase knowledge, attitudes, intentions, and uptake of the vaccine. This study is one of the first to use a multi-method evaluation to assess mHealth interventions to increase HPV vaccination."

"We found that HPVVaxFacts improved HPV vaccine initiation rates among children at significantly higher rates if their parents were in the HPVVaxFacts compared to the nutrition program."

"We were able to retain 75% in the HPV program and 94% in the nutrition program; however, we are unsure if the nutrition site was viewed but 80% viewed HPVVaxFacts."

22-ii) Highlight unanswered new questions, suggest future research

Highlight unanswered new questions, suggest future research.

	1	2	3	4	5	
subitem not at all important	○	○	○	○	◉	essential

Clear selection

Does your paper address subitem 22-ii?

Copy and paste relevant sections from the manuscript (include quotes in quotation marks "like this" to indicate direct quotes from your manuscript), or elaborate on this item by providing additional information not in the ms, or briefly explain why the item is not applicable/relevant for your study

"This study's results lay the foundation for a full-randomized control trial to test the efficacy of HPVVaxFacts being offered pre-clinic visit to increase HPV vaccine initiation and completion rates. Future studies can explore the mediating (e.g., provider recommendation and communication) and moderating factors for successful intervention effects with large sample size."

20) Trial limitations, addressing sources of potential bias, imprecision, and, if relevant, multiplicity of analyses

20-i) Typical limitations in ehealth trials

Typical limitations in ehealth trials: Participants in ehealth trials are rarely blinded. Ehealth trials often look at a multiplicity of outcomes, increasing risk for a Type I error. Discuss biases due to non-use of the intervention/usability issues, biases through informed consent procedures, unexpected events.

	1	2	3	4	5	
subitem not at all important	○	○	○	○	◉	essential

Clear selection

Does your paper address subitem 20-i? *

Copy and paste relevant sections from the manuscript (include quotes in quotation marks "like this" to indicate direct quotes from your manuscript), or elaborate on this item by providing additional information not in the ms, or briefly explain why the item is not applicable/relevant for your study

"Second, some parents may not have participated due to mobile app literacy issues or not having a mobile app, which should be explored in future studies. However, we have begun to take steps to improve inclusion through the provision of HPVVaxFacts in Spanish. Third, this study had a small sample size which may have impacted our ability to detect a difference for some of the study outcomes (e.g., attitudes, intention) and if those outcomes are a result of clinic demographics and protocol. Also, the small number of parents recruited at clinic site two made it impossible to assess whether clinic-level differences influenced results. Fourth, there were updates on the app during the feasibility study; however, study implementation including recruitment and retention improved over time. Fifth, because parents agreed to be in the study and the invite mentioned HPV vaccination, parents may be more engaged or interested in receiving information. This should be explored in a larger study.

Sixth, some parents were lost due to their child already being vaccinated during study participation and study logistics, in which we could not recruit additional participants due to study duration ending at both clinics. However, this reflects EHR interoperability, a commonly cited issue confirmed by clinic staff [63]. Seventh, we only studied the impact of HPVVaxFacts on initiation rates. The impact on completion rates (i.e. two doses of HPV vaccine among children under 15 and three doses above 15 years old) [64], will be explored in a future study as we are exploring reasons for non-compliance and informational needs of parents to inform the update of messaging on the mobile web app to be more comprehensive."

"Eighth, some mobile web app components (i.e., average session length) were not able to be tracked due to financial constraints. However, it is currently being updated to address this limitation. Last, it is possible that the attention control of nutrition education could have had an effect on parents to accept and/or obtain the initial dose of the HPV vaccination for their child."

21) Generalisability (external validity, applicability) of the trial findings

NPT: External validity of the trial findings according to the intervention, comparators, patients, and care providers or centers involved in the trial

21-i) Generalizability to other populations

Generalizability to other populations: In particular, discuss generalizability to a general Internet population, outside of a RCT setting, and general patient population, including applicability of the study results for other organizations

	1	2	3	4	5	
subitem not at all important	○	○	○	○	●	essential

Clear selection

Does your paper address subitem 21-i?

Copy and paste relevant sections from the manuscript (include quotes in quotation marks "like this" to indicate direct quotes from your manuscript), or elaborate on this item by providing additional information not in the ms, or briefly explain why the item is not applicable/relevant for your study

"First, this study was conducted in two pediatric urban clinics in Tennessee. Majority of the sample was female and White. Collectively, this may limit generalizability to other populations including those living in different geographical regions and settings along with racial and ethnic groups."

"Seventh, for the interviews, there may be potential for recall bias which increases due to the variable time period between parent participation and the interview due to the demand for study logistics."

21-ii) Discuss if there were elements in the RCT that would be different in a routine application setting

Discuss if there were elements in the RCT that would be different in a routine application setting (e.g., prompts/reminders, more human involvement, training sessions or other co-interventions) and what impact the omission of these elements could have on use, adoption, or outcomes if the intervention is applied outside of a RCT setting.

	1	2	3	4	5	
subitem not at all important	○	○	○	○	◉	essential

Clear selection

Does your paper address subitem 21-ii?

Copy and paste relevant sections from the manuscript (include quotes in quotation marks "like this" to indicate direct quotes from your manuscript), or elaborate on this item by providing additional information not in the ms, or briefly explain why the item is not applicable/relevant for your study

"For protocol application, improving the study logistics was commonly mentioned by both parents and providers though not reflective of the actual mobile web app use. This supports the idea that this tool alone could be applied in real-world settings with less human involvement, a major advantage for sustainability."

OTHER INFORMATION

23) Registration number and name of trial registry

Does your paper address CONSORT subitem 23? *

Copy and paste relevant sections from the manuscript (include quotes in quotation marks "like this" to indicate direct quotes from your manuscript), or elaborate on this item by providing additional information not in the ms, or briefly explain why the item is not applicable/relevant for your study

"The Institutional Review Board of Meharry Medical College (Protocol Number: 18-12-890) approved this study as expedited, and the study was registered at ClinicalTrials.gov (NCT# NCT04380623)."

24) Where the full trial protocol can be accessed, if available

Does your paper address CONSORT subitem 24? *

Cite a Multimedia Appendix, other reference, or copy and paste relevant sections from the manuscript (include quotes in quotation marks "like this" to indicate direct quotes from your manuscript), or elaborate on this item by providing additional information not in the ms, or briefly explain why the item is not applicable/relevant for your study

The full trial protocol is not available as this is a pilot study in progress.

25) Sources of funding and other support (such as supply of drugs), role of funders

Does your paper address CONSORT subitem 25? *

Copy and paste relevant sections from the manuscript (include quotes in quotation marks "like this" to indicate direct quotes from your manuscript), or elaborate on this item by providing additional information not in the ms, or briefly explain why the item is not applicable/relevant for your study

"This work was supported by the National Cancer Institute of the National Institutes of Health Award Number: K01CA237748-01), and the National Center for Advancing Translational Sciences (NCATS) Clinical Translational Science Award (CTSA) (award number: 5UL1TR0002243). The funding sponsors had no role in the design and conduct of the study, collection, management, analysis, or interpretation of the data; preparation, review, or approved of the manuscript or decision to submit the manuscript for publication."

X27) Conflicts of Interest (not a CONSORT item)

X27-i) State the relation of the study team towards the system being evaluated

In addition to the usual declaration of interests (financial or otherwise), also state the relation of the study team towards the system being evaluated, i.e., state if the authors/evaluators are distinct from or identical with the developers/sponsors of the intervention.

	1	2	3	4	5	
subitem not at all important	○	○	○	○	◉	essential

Clear selection

Does your paper address subitem X27-i?

Copy and paste relevant sections from the manuscript (include quotes in quotation marks "like this" to indicate direct quotes from your manuscript), or elaborate on this item by providing additional information not in the ms, or briefly explain why the item is not applicable/relevant for your study

"...The authors would also like to thank 233 analytics who developed and maintained the intervention."

About the CONSORT EHEALTH checklist

As a result of using this checklist, did you make changes in your manuscript? *

- (selected) yes, major changes
- yes, minor changes
- no

What were the most important changes you made as a result of using this checklist?

Adding additional context in each section to increase clarity based on checklist.

How much time did you spend on going through the checklist INCLUDING making changes in your manuscript *

We spent 18 hours going through the checklist.

?

As a result of using this checklist, do you think your manuscript has improved? *

- (x) yes
- ( ) no
- ( ) Other:

Would you like to become involved in the CONSORT EHEALTH group?

This would involve for example becoming involved in participating in a workshop and writing an "Explanation and Elaboration" document

- ( ) yes
- (x) no
- ( ) Other:

Clear selection

Any other comments or questions on CONSORT EHEALTH

none.

Your answer must have a minimum of 25 characters.

STOP - Save this form as PDF before you click submit

To generate a record that you filled in this form, we recommend to generate a PDF of this page (on a Mac, simply select "print" and then select "print as PDF") before you submit it.

When you submit your (revised) paper to JMIR, please upload the PDF as supplementary file.

Don't worry if some text in the textboxes is cut off, as we still have the complete information in our database. Thank you!

Final step: Click submit !

Click submit so we have your answers in our database!

Submit

Clear form

Never submit passwords through Google Forms.

This content is neither created nor endorsed by Google. - Contact form owner - Terms of Service - Privacy Policy

Does this form look suspicious? Report

Google Forms
