# Supplementary material for: A Multimethod Evaluation to Assess Feasibility, Acceptability, and Preliminary Efficacy of HPVVaxFacts, a Tailored Mobile Web App, for Parents With Unvaccinated Children: Pilot 2-Arm Randomized Controlled Trial
Source: JMIR Cancer. 2026 Sep 9;12:e78910. doi: 10.2196/78910 (PMC13557614; doi:10.2196/78910)
Supplement: Supplementary file 3 [file cancer-v12-e78910-s002.docx]

| **Table S1.** Qualitative Interviewee Socio-demographics, n=19. | | |
| --- | --- | --- |
| Parent Characteristics | HPVVax (n=9) | Nutrition (n=10) |
| Race (n, %) |  |  |
| White | 9 (100) | 7 (70) |
| African American, African, or Afro  Caribbean | 0 (0) | 1 (10) |
| American Indian/Alaskan Native | 0 (0) | 0 (0) |
| Latino or Spanish Origin | 0 (0) | 2 (20) |
| Native Hawaiian/Other Pacific Islander | 0 (0) | 0 (0) |
| Prefer not to answer | 0 (0) | 0 (0) |
| Education (n, %) |  |  |
| GED or High School Diploma | 0 (0) | 3 (30) |
| Associate’s degree | 4 (44) | 1 (10) |
| Some college | 0 (0) | 2 (20) |
| Bachelor’s Degree | 5 (56) | 3 (30) |
| Master’s Degree | 0 (0) | 1 (10) |
| Doctoral/Professional Degree | 0 (0) | 0 (0) |
| Sex (n, %) |  |  |
| Male | 0 (0) | 0 (0) |
| Female | 9 (100) | 10 (100) |
| Income (n, %) |  |  |
| Less than $20,000 | 0 (0) | 1 (10) |
| $20,001 to $40,000 | 1 (12) | 2 (20) |
| $40,001 to $60,000 | 2 (22) | 1 (10) |
| $60,001 to $80,000 | 2 (22) | 4 (40) |
| Over $80,000 | 4 (44) | 2 (20) |
| Did not want to answer | 0 (0) | 0 (0) |
| Age (µ, SD) | 38.9 (5) | 41.2 (4.7) |
